# Ubiquitin E3 ligase KPC1 governs mesenchymal metastatic melanoma reprogramming via proteasomal degradation of ZEB1

**DOI:** 10.1038/s41419-025-08262-z

**Published:** 2025-12-22

**Authors:** Yusuke Nakano, Matias A. Bustos, Kelly K. Chong, Yoshinori Hayashi, Aaron Ciechanover, Dave S. B. Hoon

**Affiliations:** 1 Department of Translational Molecular Medicine, Saint John’s Cancer Institute at Providence Saint John’s Health Center, Santa Monica, California USA; 2 The Rappaport Faculty of Medicine and Research Institute, and the Rappaport Technion Integrated Cancer Center (R-TICC), Technion – Israel Institute of Technology, Haifa, 3109601 Israel

**Keywords:** Metastasis, Melanoma, Ubiquitylation

## Abstract

Metastatic melanoma (MM) displays remarkable phenotypic plasticity, allowing tumor cells to transition reversibly between proliferative and mesenchymal (MES)-like states. This dynamic switching is strongly associated with therapeutic resistance and poor prognosis. Although transcriptional and epigenetic mechanisms driving these transitions have been extensively studied, the role of post-translational regulation, particularly the ubiquitin–proteasome system, remains poorly understood. Here, we identify the ubiquitin E3 ligase RNF 123 (KPC1) as a key post-translational suppressor of MES reprogramming in MM. Integrative analyses of bulk and single-cell transcriptomic datasets revealed that *KPC1* expression is inversely correlated with the expression of core mesenchymal markers such as *ZEB1*, *CDH2*, and *AXL*, and positively associated with epithelial and melanocytic lineage genes, including CDH1 and MITF. Deconvolution of TCGA-SKCM RNA-seq data confirmed that this inverse correlation is specific to malignant melanoma cells and strongest in tumors enriched for mesenchymal gene signatures. Single-cell trajectory and enrichment analyses further demonstrated that decreasing *KPC1* expression accompanies MES-like switch. Mechanistically, KPC1 binds and promotes the ubiquitination and proteasomal-mediated degradation of ZEB1, thereby suppressing cadherin switching and cell motility. Loss of KPC1 in melanoma cells prevented ZEB1 proteasomal-mediated degradation, increased expression of mesenchymal markers, and enhanced MM cells migration. Clinically, low KPC1 protein levels were associated with increased expression of ZEB1 and CDH2 and poorer overall survival. Furthermore, combined assessment of KPC1, ZEB1, and CDH2 expression improved patient stratification, suggesting the potential utility of multi-marker signatures for prognostic modeling. These findings establish KPC1 as a central post-translational regulator of melanoma cell state plasticity through targeted degradation of ZEB1. This study highlights a novel mechanism regulating MES-like transition and highlights KPC1 as a potential theragnostic target in MM.

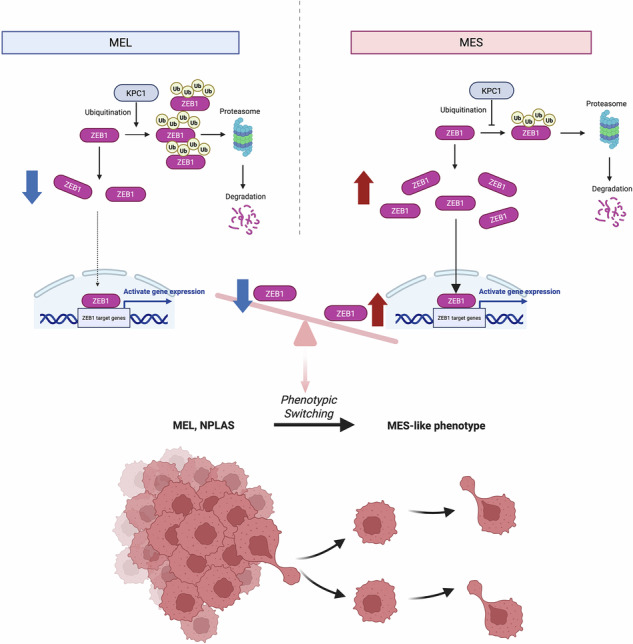

## Introduction

Cutaneous melanoma is a highly aggressive malignancy, particularly in patients with metastatic disease [1]. Melanoma incidence has been steadily increasing in Australia, Western Europe, and the United States [1–3]. The current first-line systemic treatment for metastatic melanoma (MM) involves immune checkpoint inhibitors (ICI) and/or targeted therapies, and more recently cell-based therapies [4–6]. The prognosis for patients with melanoma tumors that has metastasized to distant sites, such as visceral organs or the brain, remains dismal, with a 5-years survival rate ~35% [7, 8]. A central biological feature underlying this aggressiveness is the phenotypic plasticity of melanoma cells which implies their capacity to reversibly switch among a melanocytic, a proliferative phenotype and an invasive, and a mesenchymal (MES)-like states [9]. These cell states transition shares transcriptional features with the epithelial-to-mesenchymal transition (EMT), including loss of differentiation markers and upregulation of mesenchymal drivers, and has been linked to enhanced motility, therapeutic resistance, and metastatic potential [10, 11]. The MES-like phenotypic state is typically marked by downregulation of the microphthalmia-associated transcription factor (MITF) and upregulation of markers such as AXL receptor tyrosine kinase (AXL) and zinc finger e-box binding homeobox1 (ZEB1) [12, 13]. However, despite detailed transcriptomic characterization, the upstream mechanisms that govern this reversible switch remains poorly defined.

Although the phenotypic plasticity of melanoma has been established, the molecular circuits regulating transitions between proliferative and MES-like states are still being elucidated [14]. Signaling cascades such as the MAPK, TGF-β, and Wnt pathways have been implicated in promoting MES features and invasiveness in melanoma cells [15–17], yet these do not fully explain the dynamic reversibility or intra-tumoral heterogeneity observed in melanoma progression. ZEB1, a key transcriptional regulator of EMT in carcinomas, also plays a prominent role in the MES state of melanoma, repressing genes and facilitating an invasive behavior [18]. However, the regulatory mechanisms controlling ZEB1 expression and protein turnover in this context are poorly defined. Recent work has revealed that melanoma cell state is governed by multi-modal regulatory programs, integrating chromatin remodeling, metabolic status, transcription factor networks, and immune signaling, all of which may influence transitions between distinct cellular phenotypes [19]. Within this framework, non-genetic mechanisms —including microRNA-mediated suppression and proteasomal degradation via ubiquitin ligases— have also emerged as important contributors to phenotype switching [9, 20, 21]. Still, the involvement of the ubiquitin–proteasome system in modulating MES associated gene expression patterns in MM remains underexplored.

Kip1 ubiquitylation-promoting complex subunit 1 (KPC1), also known as RNF123, is an E3 ubiquitin ligase that targets the NF-κB precursor protein p105 for proteasomal processing, thereby modulating inflammatory and survival pathways [22, 23]. Beyond its canonical function in NF-κB regulation, KPC1 has been implicated in diverse tumor-suppressive roles across multiple cancer types, including glioblastoma, breast, and prostate cancer, by controlling cell cycle regulators and affecting EMT components [24, 25]. For instance, it has been shown to mediate ubiquitination of p27 Kip1 and Vimentin, suggesting its involvement in cell cycle control and inhibition of mesenchymal programs [26, 27]. However, no prior studies have examined whether KPC1 modulates the MES transcriptional program or phenotype switching in MM.

In this study, we investigated the role of KPC1 in regulating phenotypic plasticity in MM. Through integrative analysis combining bulk and single-cell transcriptomics, multiplex immunofluorescence (mIF), and functional assays, we demonstrated that KPC1 negatively regulates the MES program by promoting the ubiquitin-dependent degradation of ZEB1 —a central driver of mesenchymal reprogramming. Downregulation of KPC1 results in ZEB1 stabilization, increased expression of MES markers, and enhanced cellular migration. Downregulation of KPC1 is also associated with poor clinical outcomes in MM patients. Our findings position KPC1 as a gatekeeper of melanoma cell state transitions and suggest that restoration or mimicking of KPC1 activity may represent a novel therapeutic approach to suppress melanoma progression and metastasis.

## Materials and methods

### Melanoma cell lines

Established MM cell lines from SJCI were attained from melanoma patients who received elective surgery (DP-0574, FD-0836, HM-0525, LP-0024, ML-0817, MH-0331, M-12, M204, VN-0326, WP-0614). The cell lines were cultured in RPMI-1640 and supplemented with 10 mM HEPES, 10% heat-inactivated fetal bovine serum (FBS) and 1% penicillin-streptomycin (complete medium). All human cell lines have been authenticated using short tandem repeat (STR) profiling within the last three years. All experiments were performed with mycoplasma-free cell lines.

### Public datasets

Bulk RNA sequencing (RNA-seq) datasets from The Cancer Genome Atlas (TCGA) [28] and Genotype-Tissue Expression (GTEx) [29] were downloaded through the website of University of California Santa Cruz (UCSC) Xena [30]. Single-cell RNA-seq (scRNA-seq) dataset of melanoma was obtained from GSE115978 [31], which includes transcriptomic profiles of 31 melanoma tumors from patients with metastatic melanoma who were treated with immune checkpoint inhibitors (ICI). The protein expression dataset of melanoma patients was obtained from PXD006003 [32], which includes proteomic profiles of advanced-stage melanoma patients undergoing tumor-infiltrating lymphocyte (TIL)-based or anti-PD1 immunotherapy, quantified using high-resolution mass spectrometry.

### Deconvolution analysis of the bulk RNA sequencing data sets

BayesPrism [33] and CODEFACS [34] deconvolution analyses were applied to the bulk RNA sequencing (RNA-seq) data set of TCGA-SKCM [30] to obtain melanoma cell-specific expression profiles. For BayesPrism, the count matrixes of messenger RNA (mRNA) expression profiles were provided as input and the raw count matrix of the single cell RNA-seq (scRNA-seq) data set from GSE115978 [31] was used as the reference gene expression profile. For CODEFACS, the mRNA expression profiles of Transcripts Per Million (TPM) format were provided as input and the same gene expression profile generated in the previous study was used as reference [34]. CODEFACS deconvolution analysis was used to get the immune cell fractions. For CODEFACS, the Leukocyte Signature Matrix 22 (LM22) signature matrix [35] was used as the reference gene expression profiles of immune cells. LM22 was downloaded from the CIBERSORTx web portal (https://cibersortx.stanford.edu/) [35].

### Pseudospatial reconstruction of single-cell transcriptomes

Pseudospatial reconstruction of single-cell transcriptomes was performed using Monocle3 algorithm [36, 37]. Cells were ordered based on genes identified through differential gene expression analysis, comparing inner and outer cell fractions. Significant genes were selected using a likelihood ratio test, with criteria of FDR < 1 × 10⁻¹⁰ and |log₂ fold-change | > 1. These genes were used as “ordering genes” to construct trajectories with Monocle3 functions (setOrderingFilter(), reduceDimension(), and orderCells()). MES marker expression across pseudospace was visualized using the plot_genes_in_pseudotime function [36, 37].

### Statistical analysis

Variations among variables were compared using either the Mann–Whitney U test, Student’s t-test, or Fisher’s exact test where applicable. Multiple groups were analyzed by one-way ANOVA followed by Tukey’s post hoc test. The Kaplan–Meier method was utilized to estimate overall survival (OS), progression-free survival (PFS) and disease-specific survival (DSS), and comparisons between survival curves were made using the Log-rank test. The statistical analyses were two-sided and performed using R software v4.4.2. A significance level of *p* < 0.05 was considered statistically significant. All experiments were performed in triplicate unless otherwise noted.

Additional M&M and uncropped western blot images can be found in Supplementary Information, Table S1 and S2, Supplementary Material files. All Western blot quantifications are provided in Figs. S7 and S8.

## Results

### KPC1 expression is inversely associated with key mesenchymal markers in MM

**Fig. 1:**
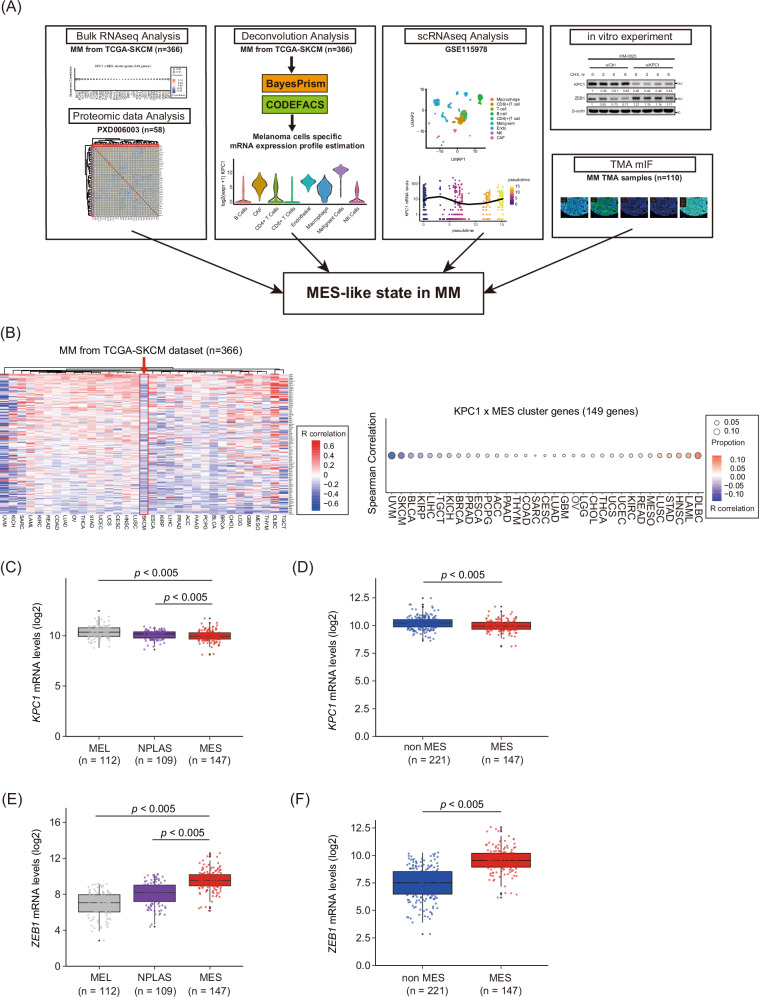
Integrative analysis reveals KPC1 downregulation during MES Switching in MM. **A** Schematic overview of multimodal approaches used to define mesenchymal (MES) state switching in metastatic melanoma (MM). **B** Left: Heatmap of Spearman correlation between *KPC1* and 149 MES signature genes across 33 TCGA tumor types; a red arrow highlights TCGA-SKCM dataset. Right: Dot plot of Spearman’s correlation values (R) between *KPC1* and MES signature genes in each tumor type; circle size indicates the fraction of significant genes negative or positive correlated with *KPC1*. **C** Box plot of *KPC1* mRNA (log2) in TCGA-SKCM samples stratified as MEL, NPLAS, or MES. **D** Box plot of *KPC1* mRNA (log2) in TCGA-SKCM samples stratified as MES or non-MES. **E** *ZEB1* mRNA (log2) in MEL, NPLAS, and MES subsets of TCGA-SKCM dataset. **F** *ZEB1* expression in non-MES versus MES subsets of TCGA-SKCM dataset. Data are presented as the mean ± standard deviation. Three-group comparisons: one-way ANOVA with Tukey’s post hoc test.

To examine the relationship between KPC1 expression and mesenchymal reprogramming in MM, we employed a five-pronged integrative strategy (Fig. 1A). As an initial step, we performed a comprehensive correlation analysis between *KPC1* mRNA levels and mesenchymal gene signatures across 33 cancer types, including samples from the TCGA-SKCM cohort. Although correlation patterns varied among tumor types, *KPC1* mRNA levels generally exhibited a negative association with MES marker genes (Figs. 1B and S1A, B). Building on a previous classification of MM into melanocytic (MEL), neural/plastic (NPLAS), MES clusters based on MES-associated gene signatures [19], we next compared *KPC1* expression across these transcriptomic-based molecular subtypes. Notably, *KPC1* expression was significantly reduced in the MES cluster (Fig. 1C, D). In contrast, *ZEB1*, *CDH2*, and *AXL* mRNA levels —canonical MES markers— showed the highest levels in the MES cluster (Fig. 1E, F; Fig. S2A, C), whereas *CDH1* and *MITF* expression were the lowest in MES cluster (Fig. S2B, D). We further stratified MM into *KPC1*-high and *KPC1*-low groups. In the low-*KPC1* group, *ZEB1*, *AXL*, and *CDH2* were significantly upregulated (Fig. S2E, F, H), while *CDH1* and *MITF* were significantly upregulated in the high-*KPC1* group (Fig. S2G, I). Correlation analyses confirmed that *KPC1* was significantly inversed correlated with *ZEB1*, *AXL*, and *CDH2* (Fig. S2J, K, M), and positively correlated with *CDH1* and *MITF* (Fig. S2L, N).

To validate these findings at the protein level, we analyzed data from the PXD006003 proteomic dataset. Consistent with the transcriptomic observations, KPC1 protein levels were inversely correlated with MES markers (Fig. S1C, D). Together, these findings support a robust inverse relationship between KPC1 expression and MES gene programs in MM at both the mRNA and protein levels.

### Deconvolution analysis reveals a negative correlation between KPC1 and genes in melanoma cells

**Fig. 2:**
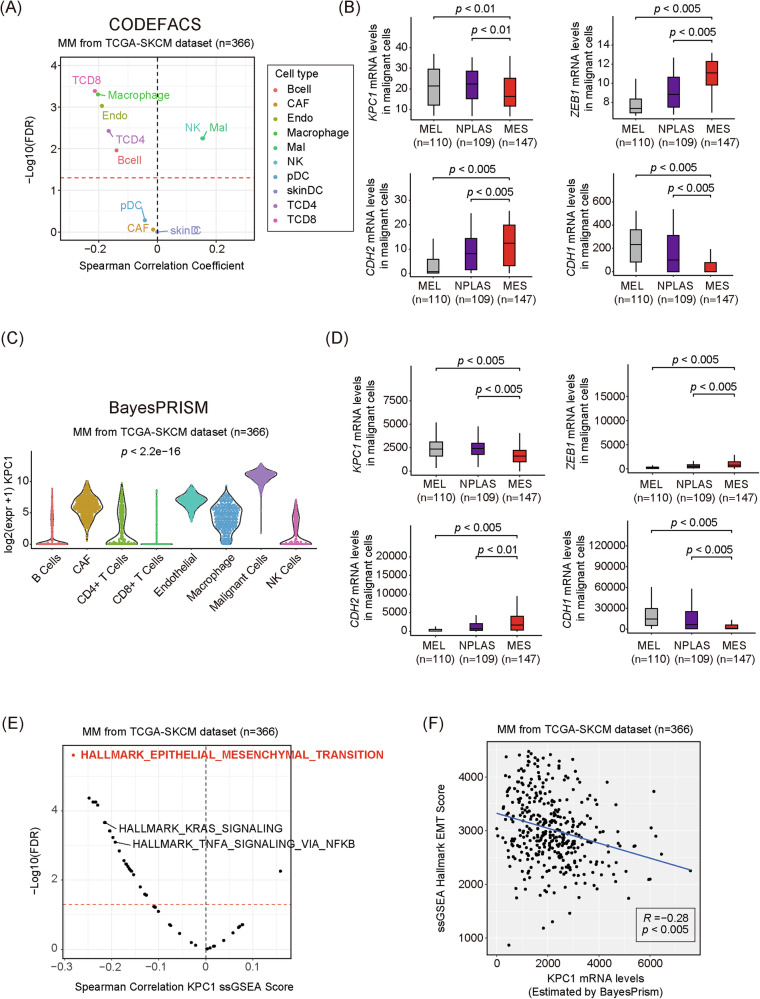
Deconvolution analysis of *KPC1* mRNA levels with MES markers and pathways in MM cells. **A** Spearman’s correlation between infiltrated immune cell fraction levels and *KPC1* mRNA levels in metastatic melanoma cells, estimated by CODEFACS. **B** Box plot of *KPC1, ZEB1, CDH2*, and *CDH1* mRNA levels in MEL, NPLAS, and MES clusters, estimated by CODEFACS. **C** Distribution of *KPC1* mRNA levels in different cell types, estimated by BayesPrism. **D** Box plot of *KPC1*, *ZEB1, CDH2*, and *CDH1* mRNA levels in MEL, NPLAS, and MES clusters, estimated by BayesPrism. **E** Spearman’s correlation between *KPC1* mRNA levels and pathway enrichment scores, calculated by ssGSEA in TCGA-SKCM metastatic melanoma samples. **F** Spearman’s correlation coefficient (R) between *KPC1* mRNA levels and EMT pathway enrichment scores, calculated by ssGSEA in TCGA-SKCM metastatic melanoma samples. Three-group comparisons: one-way ANOVA with Tukey’s post hoc test.

Bulk RNA-seq data contains a mixture of the expression profiles from various cell types, making it potentially insufficient to clearly determine that the relationship between *KPC1* expression and MES marker genes occur specifically in tumor cells. To address this limitation, deconvolution analysis was performed on MM samples from the TCGA-SKCM RNA-seq dataset using CODEFACS and BayesPrism pipelines. The deconvolution analysis with CODEFACS identified ten distinct cell types (Fig. 2A). The highest *KPC1* mRNA levels were observed in the malignant melanoma cell fraction (Fig. 2A). Among the MEL, NPLAS, and MES clusters, *KPC1* expression was the lowest in the MES cluster (Fig. 2B). In contrast, *ZEB1* and *CDH2* exhibited the highest expression levels in the MES cluster, whereas *CDH1* expression was also reduced (Fig. 2B). Similarly, BayesPrism-based deconvolution analysis also confirmed that malignant melanoma cells exhibited the highest *KPC1* expression levels (Fig. 2C). Among the three clusters, BayesPrism-based analysis yielded consistent results, showing that *KPC1* expression was lowest in the MES cluster (Fig. 2D). Conversely, *ZEB1* and *CDH2* displayed the highest expression levels in the MES cluster, while *CDH1* expression was also lower in this group (Fig. 2D). Subsequently, single-sample gene set enrichment analysis (ssGSEA) was conducted using the deconvoluted melanoma cell profiles from MM samples obtained from the TCGA-SKCM dataset to explore signaling pathways associated with *KPC1*. These analyses revealed that *KPC1* levels in MM cells were negatively correlated with the ssGSEA hallmark EMT pathway score (Fig. 2E, F). In summary, these findings indicate that *KPC1* expression in MM cells is negatively correlated with MES marker genes, suggesting a link between *KPC1* levels and the mesenchymal status in MM cells.

### Single-cell RNA-seq analysis highlights the association between KPC1 and MES marker genes in MM

**Fig. 3:**
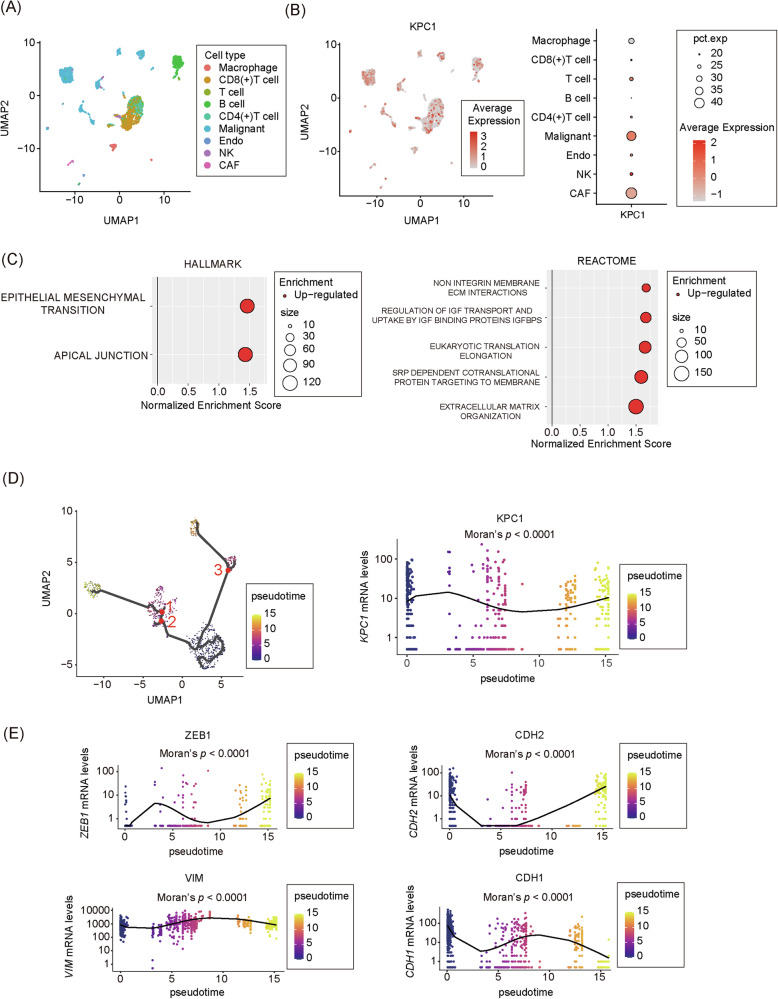
Single-cell RNA-seq analysis of *KPC1* mRNA levels and its association with MES genes in MM. **A** t-SNE plot showing the clustering of nine distinct cell types based on single-cell RNA-seq data from metastatic melanoma (GSE115978). Colors represent post hoc annotation of single-cell profiles. **B** Distribution of *KPC1* mRNA levels across cell types. Left: Heatmap showing *KPC1* expression in different cell types. Right: Dot plots illustrate *KPC1* mRNA levels in melanoma cells compared to other cell types. Single-cell expression profiles were re-clustered using Seurat (v4.0.5). **C** Pathway enrichment analysis of melanoma cells with low *KPC1* expression group. Left: Hallmark enrichment pathway. Right: REACTOME enrichment pathway. **D** Left: Trajectory analysis was conducted using single-cell RNA-seq data to examine the relationship between *KPC1* expression and MES enrichment. Right: The analysis illustrates the progression of MES and the corresponding changes in *KPC1* expression. Statistical significance of the trajectory was evaluated using Moran’s I. **E** Expression patterns of mesenchymal marker genes, including MES-promoting factors (ZEB1, CDH2, and VIM) and MES-suppressing factors (CDH1), were evaluated along the trajectory of MES enrichment. Statistical significance of the trajectory was evaluated using Moran’s I.

To further investigate the association between KPC1 and mesenchymal marker genes in MM cells, we performed a detailed analysis using scRNA-seq data from MM. scRNA-seq analysis identified nine distinct cell types (Fig. 3A), where malignant melanoma cells showed the highest *KPC1* expression (Fig. 3B). Additionally, pathway analysis revealed that MES related pathways were significantly enriched in the *KPC1* low-expression group (Fig. 3C). To explore the relationship between KPC1 and MES in greater detail, trajectory analysis was conducted using the scRNA-seq data. Importantly, there was an MES enrichment followed by a decrease in *KPC1* expression (Fig. 3D). Furthermore, MES-promoting factors such as *ZEB1*, *CDH2*, and *VIM* were upregulated with MES enrichment, whereas MES-suppressing factors like *CDH1* were downregulated as MES became more enriched (Fig. 3E). In summary, these findings provide strong evidence that *KPC1* expression is inversely associated with MES enrichment in MM. This is accompanied by the upregulation of MES-promoting genes and the downregulation of MES-suppressing genes, suggesting a dynamic interplay between KPC1 expression and the transition to a MES-like state in MM cells.

### KPC1 silencing drives post-transcriptional stabilization of ZEB1 and MES reprogramming

**Fig. 4:**
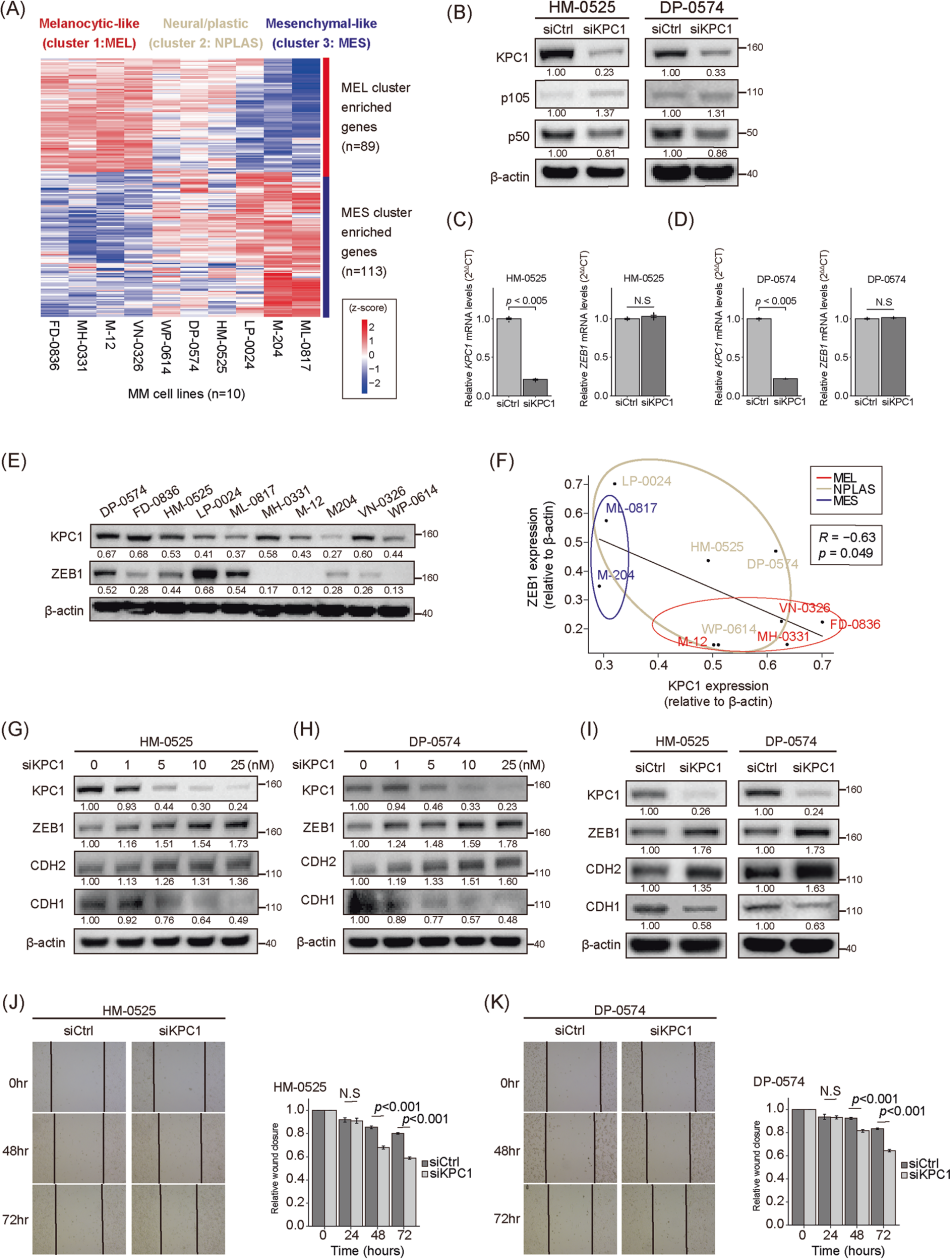
KPC1 knockdown drives mesenchymal reprogramming in MM cell lines. **A** Heatmap showing relative expression (z-score) of 89 melanocytic-like (MEL) and 113 mesenchymal-like (MES) signature genes across ten metastatic melanoma (MM) cell lines, clustered into MEL, neural/plastic (NPLAS), and MES subtypes. **B** Western blot images show KPC1, p105, and p50 protein levels following siRNA-mediated silencing of KPC1 (siKPC1) in HM-0525 and DP-0574 cell lines compared to control conditions (siCtrl). **C**, **D** qRT-PCR analysis of *KPC1* and *ZEB1* in HM-0525 (**C**) and DP-0574 (**D**) cells transfected with siCtrl or siKPC1. **E** Western blot images show KPC1 and ZEB1 protein levels in various MM cell lines. **F** Scatter plot showing the correlation levels between endogenous KPC1 and ZEB1 protein levels (relative to β-actin) across the ten MM cell lines; subtypes colored as in (**A**), Pearson’s correlation coefficient (r) and p-value indicated. **G**, **H** Western blot images of KPC1, ZEB1, CDH2, and CDH1 following siKPC1 at varying concentrations (1, 5, 10, 25 nM) in HM-0525 and DP-0574 cell lines. **I** Western blot images of KPC1, ZEB1, CDH2, and CDH1 protein levels following siKPC1 vs siCtrl in HM-0525 and DP-0574 cell lines. **J**, **K** Wound healing assays using HM-0525 and DP-0527 cells transfected with siCtrl or siKPC1. The migrated distance was quantified by measuring the difference at 0, 24, 48, and 72 h and was normalized to 0-hour timepoint. Data are presented as the mean ± standard deviation. qRT-PCR data were analyzed by two-tailed unpaired Student’s t-test. Wound healing data were analyzed by two-way repeated-measures ANOVA with Bonferroni post-hoc correction for multiple comparisons. These data represent three independent experiments, each conducted in triplicate. N.S. not significant.

To investigate the functional consequences of KPC1 downregulation, we examined MM cell lines previously classified into MEL, NPLAS, and MES transcriptomic subtypes [19] (Fig. 4A). Our focus was on determining whether p65 or KPC1-mediated regulation of p50 levels had any effect on ZEB1 transcriptional regulation. Silencing of canonical NF-κB pathway component p65 did not affect ZEB1 expression at either the mRNA or protein level (Fig. S3A–D). *KPC1*-knockdown led to robust depletion of KPC1, accumulation of its canonical substrates p105, and a decrease in p50 (Fig. 4B), consistent with our previous reports [25], but *ZEB1* mRNA levels remained unchanged (Fig. 4C, D), confirming that NF-κB signaling is dispensable for ZEB1 transcriptional regulation. In contrast, baseline KPC1 protein inversely correlated with ZEB1 abundance across ten MM cell lines (Fig. 4E, F). *KPC1-* knockdown induced a dose-dependent stabilization of ZEB1 and CDH2, along with reduced CDH1 expression, indicative of a cadherin switch (Figs. 4G–I and S3E, F). Functionally, KPC1-deficient cells showed enhanced migratory activity in wound-healing assays (Fig. 4J, K). In a previous study, VIM was reported to be a substrate for KPC1 [27]; however, VIM protein levels were unchanged in MM cell lines with KPC1 downregulation (Fig. S3H). MITF is a key transcriptional factor associated with a melanocytic lineage program [19]. Based on the MES-like phenotype switching, we evaluated MITF levels to determine whether KPC1 knockdown in MM cell lines. Consistently with a MES-like phenotype switching, MITF decreased at both the mRNA and protein levels (Fig. S3G, H). These data support the role for KPC1 in suppressing MES reprogramming via post-transcriptional regulation of ZEB1 and maintaining MITF levels.

Next, we examined whether externally TGF-β induced MES-like switching changes the KPC1 abundance in MM cell lines. The TGF-β induced MES-like state in MM cell lines was characterized by a spindle morphology, ZEB1/CDH2 upregulation and CDH1 downregulation, together with a decrease in KPC1 protein (Fig. S4A, B). These results suggest that TGF-β induced MES-like switching may be a potential trigger of KPC1 downregulation, which consequently initiates a MES-like phenotype switching in MM.

### KPC1 mediates ubiquitin-dependent proteasome turnover of ZEB1 in MM cells

**Fig. 5:**
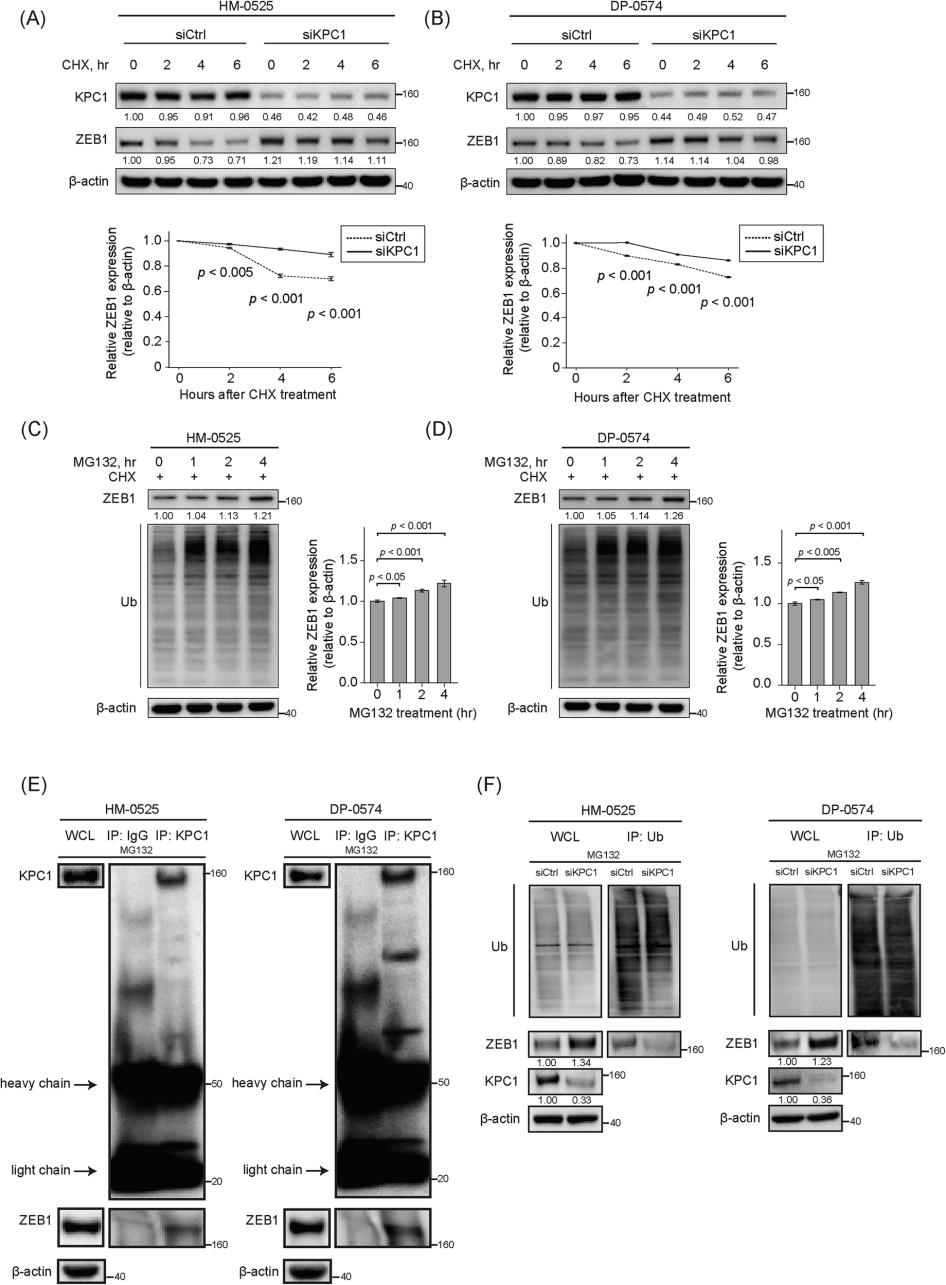
KPC1 controls ZEB1 protein turnover via ubiquitin-proteasome pathways. **A**, **B** Cycloheximide (CHX) chase in HM-0525 (**A**) and DP-0574 (**B**) cells transfected with control siRNA (siCtrl) or KPC1 siRNA (siKPC1). Cells were treated with CHX (50 µg/mL) for 0, 2, 4, or 6 h. Upper panels show representative Western blots images for KPC1 and ZEB1; β-actin is loading control. Lower panels plot mean ± SD of ZEB1/β-actin from three independent experiments. **C**, **D** Proteasome inhibition in CHX-treated cells. HM-0525 (**C**) and DP-0574 (**D**) cells were cotreated with CHX (50 µg/mL) and MG132 (10 µM) for 0, 1, 2, or 4 h. Left, Western blots images for ZEB1 (top), ubiquitin (Ub; middle), and β-actin (bottom). Right, quantification of ZEB1/β-actin (mean ± SD, n = 3); statistical comparisons versus 0 h by one-way ANOVA with Dunnett’s post hoc test. **E** Immunoprecipitation of endogenous KPC1. HM-0525 and DP-0574 cells were treated with MG132 (10 µM, 4 h), lysed, and subjected to IP with anti-KPC1 or control IgG. Whole-cell lysate (WCL), IgG IP, and KPC1 IP fractions were Western-blotted for KPC1 (inset) and ZEB1; β-actin is loading control. Heavy-chain and light-chain bands of the antibody used in IP are indicated. **F** Ubiquitin-Trap assay for ZEB1 ubiquitination. DP-0574 and HM-0525 cells transfected with siCtrl or siKPC1 were treated with MG132 (10 µM, 4 h). Ubiquitin-binding beads captured ubiquitinated proteins from cleared lysates; eluates and WCL were analyzed by Western blot using for total Ubiquitin (top), ZEB1 (middle) and KPC1 (bottom) protein levels; β-actin served as loading control.

Based on the ZEB1 protein regulatory effect mediated by KPC1 in MM cell lines, we next assessed whether KPC1 controls ZEB1 protein turnover. Cycloheximide (CHX) chase assays showed that *KPC1*- knockdown delayed ZEB1 degradation over 6 h, indicating that KPC1 loss stabilizes ZEB1 (Fig. 5A, B). Proteasome inhibition using MG132 further confirmed that ZEB1 is degraded via the ubiquitin–proteasome system. MG132 treatment increased ZEB1 levels and induced accumulation of polyubiquitinated ZEB1 species (Fig. 5C, D). Co-immunoprecipitation revealed that endogenous KPC1 interacted with endogenous ZEB1 in MM cells (Fig. 5E). In immunoprecipitation assay using ubiquitin-trap beads, *KPC1*- knockdown was linked to a reduced level of ubiquitinated-ZEB1 (Fig. 5F). Together, these findings demonstrated that KPC1 promotes the ubiquitin-dependent proteasomal degradation of ZEB1 in MM cells and suggested that KPC1 may represent an E3-ligase mediating ZEB1 ubiquitination.

Then, we wondered whether ZEB1 transcription factor controls KPC1 protein levels in a feedback loop. To address that MM cell lines with high endogenous levels of ZEB1 and low endogenous levels of KPC1 were treated with siRNA targeting ZEB1. Surprisingly, ZEB1 downregulation did not affect the protein levels of KPC1 (Fig. S4C–E). These results suggest that KPC1 protein levels are not affected by ZEB1 downregulation to maintain the MES-like phenotypes in MM cell lines.

### KPC1 loss correlates with MES marker upregulation and poor prognosis in MM

**Fig. 6:**
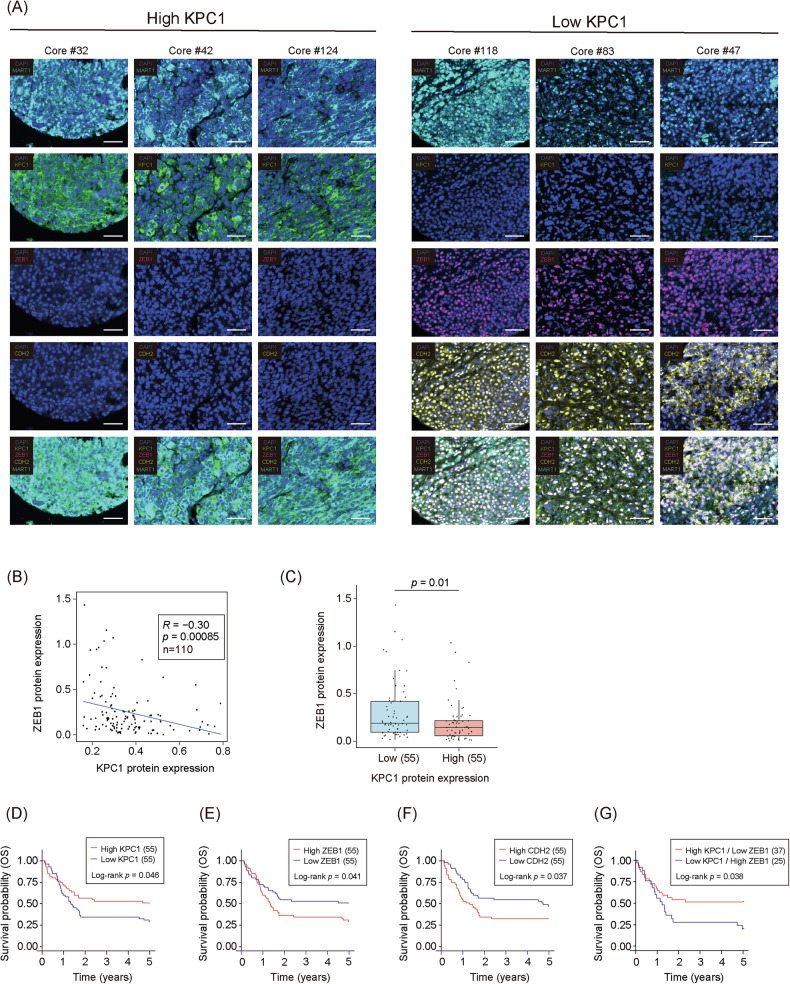
Multiplex immunofluorescence and clinical correlations of KPC1, ZEB1, and CDH2 protein levels in MM TMA. **A** Representative multiplex immunofluorescence images from TMA cores stratified by KPC1 protein expression (“High” vs. “Low”; *n* = 3 cores per group). Rows show single-channel DAPI, MART1 (cyan), KPC1 (green), ZEB1 (magenta), CDH2 (yellow), and the merged four-color overlay. Scale bars, 100 µm. **B** Scatter plot of KPC1 versus ZEB1 protein levels (*n* = 110 patients). Spearman’s correlation coefficient (R) and two-tailed p-value are indicated. **C** Box plots comparing ZEB1 protein expression between Low KPC1 (*n* = 55) and High KPC1 (*n* = 55) patient groups. **D**–**G** Kaplan–Meier analyses for overall survival (OS) when considering the protein levels of (**D**) KPC1, (**E**) ZEB1, (**F**) CDH2, and (**G**) combined KPC1/ZEB1 expression subgroups in 110 MM patients. Numbers in parentheses denote group sizes. Log-rank test *p*-values are indicated.

To assess the relationship between KPC1 downregulation and MES marker expression in tumors from MM patients, a TMA was stained for MART1, KPC1, ZEB1 and CDH2 by mIF. 110 cores from MM patients were evaluated for staining after quality control. MM tumors with high KPC1 protein showed robust KPC1 signals that were accompanied by low levels of ZEB1 and CDH2; conversely, MM tumors with low KPC1 exhibited low KPC1 protein levels, but markedly increased ZEB1 and CDH2 protein levels (Fig. 6A). Quantitative image analysis across all cores revealed a significant inverse correlation between KPC1 and ZEB1 protein levels (Fig. 6B), and ZEB1 abundance was higher in the KPC1-low group compared to KPC1-high group (Fig. 6C). We next evaluated the prognostic impact of KPC1, ZEB1 and CDH2 protein expression using the clinical annotations for the MM patients included in the TMA. MM patients with low KPC1 expression experienced markedly poorer overall survival (OS, Fig. 6D). Likewise, high ZEB1 or high CDH2 protein levels in the tumor cells (MART-1^+^) were associated with a worse prognosis (Fig. 6E, F). MM patients having low KPC1 expression and high ZEB1 protein levels exhibited a significantly poorer prognosis (Fig. 6G). Together, these data demonstrated that KPC1 downregulation in MM correlates with MES marker upregulation. KPC1, ZEB1, and CDH2 carry independent prognostic value in MM patients.

### Role of KPC1 as a biomarker in SKCM and UVM

As previously described, *KPC1* mRNA levels are downregulated in MM tissue [25]. In this exploratory analysis across solid tumors using TCGA dataset, we analyzed data from 33 tumor types and 30 corresponding normal tissues datasets. We demonstrated that *KPC1* is highly expressed in tumor tissues compared to normal tissues across various cancer types (Fig. S5A). Additionally, patients were stratified based on *KPC1* expression in multiple cancers, including MM. Of clinical relevance, low *KPC1* expression was associated with significant worse OS, disease-specific survival (DSS), and progression-free survival (PFS), in both SKCM and UVM (Fig. S5B–D). These findings suggested that KPC1 has the potential to serve as a biomarker context dependent on specific cancer types. To determine whether expression of mesenchymal markers stratifies patient outcome in MM, we analyzed OS using TCGA-SKCM dataset according to *ZEB1*, *CDH2* and *CDH1* mRNA levels. Patients with high *ZEB1* had significantly shorter OS than those with low *ZEB1* (Fig. S6A). Similarly, high *CDH2* expression predicted worse OS compared with low *CDH2* (Fig. S6A), and low *CDH1* was associated with inferior OS relative to high *CDH1* (Fig. S6A). We next examined combined signatures: patients with low *KPC1* and high *ZEB1* fared markedly worse than those with high *KPC1* and low *ZEB1* (Fig. S6B), and inclusion of *CDH2* further refined risk stratification, with the low *KPC1*/high *ZEB1*/high *CDH2* group showing the poorer OS (Fig. S6B). These data demonstrate that elevated *ZEB1* and *CDH2*, as well as reduced *CDH1*, portend worse clinical outcomes in MM, and that combined assessment of *KPC1*, *ZEB1* and *CDH2* expression can further improve prognostic precision.

## Discussion

The present study identifies KPC1 as a key negative regulator of MES reprogramming in MM. Through integrative transcriptomic, proteomic, and functional analyses, we demonstrated that KPC1 expression is inversely associated with mesenchymal markers such as ZEB1 and CDH2, and AXL, and positively correlated with epithelial regulators including CDH1 and MITF. Mechanistically, KPC1 promotes the ubiquitination and proteasomal degradation of ZEB1, thereby limiting cadherin switching, cell migration, and the activation of a mesenchymal transcriptional program. These findings establish KPC1 as a post-translational gatekeeper of MES-like cell state in MM.

The results of this study extends the current mechanistic models of transcriptional and epigenetic control of melanoma phenotypic switching [38, 39], to demonstrate that ubiquitin–proteasome–mediated regulation of ZEB1 protein stability represents a critical and underexplored layer of control. This mechanism explains how rapid and reversible phenotypic transitions may occur, potentially independently of immediate alterations in mRNA levels. Intriguingly, while KPC1 is known to modulate NF-κB signaling via p105 processing to p50 [22], our findings indicate that KPC1-mediated suppression of ZEB1, a key driver of the MES state, occurs independently of the canonical NF-κB p65/p50 pathway. This is evidenced by the lack of ZEB1 alteration upon p65 silencing, suggesting a distinct, direct regulatory role for KPC1 in controlling melanoma plasticity via ZEB1 that is separate from its impact on NF-κB signaling. Furthermore, our findings suggest that KPC1 activity is intrinsically linked to the established MEL (MITF-high/AXL-low) versus MES (MITF-low/AXL-high) lineage states. Specifically, the higher *KPC1* expression in tumors with melanocytic signatures and lower expression in MES-enriched tumors positions KPC1 as a potential upstream regulator or a key stabilizing factor for the melanocytic phenotype, possibly by suppressing ZEB1-driven mesenchymal programs that antagonize MITF expression or function. Conversely, a decrease in KPC1 levels may lower the threshold for cells to switch towards a ZEB1-high/MITF-low mesenchymal state, a transition strongly associated with increased invasiveness and therapy resistance. In our system, KPC1 loss was accompanied by reduced MITF at both transcript and protein levels while vimentin remained unchanged, supporting a lineage-shift interpretation rather than vimentin-mediated effects. Consistent with this view, exogenous induction of a MES-like program was associated with lower KPC1 in MM cell lines, suggesting that phenotype-switching cues can modulate KPC1 and thereby favor ZEB1 stabilization. In line with this framework, our prior work showed that upregulation of miR-155 reduces KPC1 in MM cells [25], and TGF-β has been reported to increase miR-155 and promote cell invasion [40], providing a plausible miR-155–mediated link between MES-like induction and reduced KPC1. Collectively, these results complement the existing models based on the dynamic interplay of known melanoma lineage-defining transcription factors like MITF, SOX10, BRN2, and AXL [10, 14], by adding KPC1 as a crucial checkpoint. Furthermore, our findings that KPC1 acts upstream to control ZEB1 stability align with and provide a novel regulatory mechanism for previous studies highlighting ZEB1 as a central mediator of invasive and therapy-resistant melanoma states [18].

Previous study by our group demonstrated that KPC1 is downregulated as melanoma patients progressed to advance stages [25]. As noted above, the downregulation of KPC1 could be explained by an upregulation of miR-155-5p in MM tumors that target *KPC1* mRNA levels [25]. From a translational perspective, restoring or mimicking KPC1 function may offer a novel means to restrict tumor progression given the association between mesenchymal states and drug resistance, immune evasion, and metastasis [11, 14]. Therefore, potential strategies aimed at pharmacologic enhancement of KPC1 expression or ligase activity, as well as targeted degradation of ZEB1 via PROTACs or other modalities, may hold therapeutic value. Additionally, the combined expression profile of KPC1 and ZEB1 may serve as a prognostic biomarker, enabling improved risk stratification in patients with MM.

While our study provides robust and reproducible findings regarding the KPC1-ZEB1 axis using siRNA-mediated knockdown in in vitro models, we acknowledge certain limitations inherent to this approach. The use of cell lines, though informative for mechanistic dissection, may not fully recapitulate the complex tumor microenvironment and heterogeneity observed in vivo. Furthermore, while siRNA offers effective target downregulation, future studies employing alternative genetic manipulation techniques could further solidify the specific role of KPC1. Therefore, subsequent investigations will be valuable to ascertain the broader physiological and pathological relevance of KPC1-mediated regulation of tumor plasticity and its implications for therapeutic responses in more complex, clinically relevant settings.

In summary, this study uncovers that KPC1 is a suppressor of MES status in MM by mediating the ZEB1 ubiquitination and proteasomal degradation. These findings contribute to a growing understanding of post-translational regulation of the ZEB1 master regulator of MES status and tumor plasticity associated with resistance and immune evasion. KPC1 could be a potential target for theragnostic target development in MM patients undergoing targeted or ICIs therapies. The MES status of melanoma may be indicative of potential non-responsiveness to certain types of therapeutic interventions thus may serve as a triaging factor to specific therapies.

## Supplementary information

Supplementary Information

Table S1

Table S2

Supplementary Materials WB uncropped

## Data Availability

Data are available in a public, open access repository. All datasets and databases utilized throught the manuscript are described in Table S1.
