# Supplementary material for: Ubiquitin E3 ligase KPC1 governs mesenchymal metastatic melanoma reprogramming via proteasomal degradation of ZEB1
Source: Cell Death Dis. 2025 Dec 22;16(1):897. doi: 10.1038/s41419-025-08262-z (PMC12722757; doi:10.1038/s41419-025-08262-z)
Supplement: Supplementary file 1 — Supplementary Information [file 41419_2025_8262_MOESM1_ESM.docx]

**Supplementary Information** for

**Ubiquitin ligase KPC1 governs mesenchymal reprogramming via proteasomal degradation of ZEB1**

Yusuke Nakano *et al*.

*Corresponding author. E-mail: [dave.hoon@providence.org](http://dave.hoon@providence.org)

**Supplementary M&M**

**RT-PCR**

RNA was extracted using the Direct-zol RNA miniprep kit (Zymo Research, Irvine, CA). The RT-qPCR was performed using a Roche LightCycler thermocycler as previously described ^1, 2^. RT-qPCR was performed in five independent biological experiments (n = 5). Primer sets used in RT-qPCR are provided in Table S1.

**Western blot assays**

Traditional western blot was performed as previously described ^1^. In brief, equal quantities of protein (25 μg) were separated on 4-20% Tris-glycine polyacrylamide gels, followed by transfer to nitrocellulose membrane (Cat#1620115, Bio-Rad, Hercules, CA) at 70 V for 1 hours at room temperature. To block nonspecific binding, membranes were incubated in blocking buffer (TBS and 0.1% Tween-20 with 5% nonfat milk powder) for 1 hour at room temperature. Subsequently, the membranes were exposed to specific primary antibodies (Ab) (KPC1 at 1:1,000 dilution, sc101122, Santa Cruz Biotechnology, Dallas, TX; ZEB1 at 1:1,000 dilution, 3396, Cell Signaling Tech, Danvers, MA; CDH1 at 1:1,000 dilution, 3195, Cell Signaling Tech, Danvers, MA; CDH2 at 1:1,000 dilution, 4061, Cell Signaling Tech, Danvers, MA; VIM at 1:1,000 dilution, 5741, Cell Signaling Tech, Danvers, MA; MITF at 1:1,000 dilution, NB100-56561, Novus Biologicals, Centennial, CO; β-actin at 1:10,000 dilution Sigma Aldrich, A5441, St. Louis, MO) in blocking buffer overnight at 4°C. Following three washing steps, the blots were incubated with suitable secondary Ab (anti-rabbit secondary Ab at 1:4,000, 7074, Cell signaling Tech, Danvers, MA; anti-mouse secondary antibody at 1:4,000, 7076, Cell signaling Tech, Danvers, MA) for 1 hour. After another round of three washing steps, chemiluminescence detection was performed using the iBright CL1500 Imaging System (Thermo Fisher Scientific, Waltham, MA) and density was quantified using ImageJ software (ver.1.54n, National Institutes of Health, Bethesda, MD) ^3^. All Western blot experiments were repeated in three independent biological experiments (n = 3), and densitometric quantification was performed across all replicates. All the uncropped western blot images were included in Supplementary Materials.

**Immunoprecipitation (IP)**

Co-IP experiments were performed as previously described ^2^. MM cell lines were washed with PBS and lysed in the immunoprecipitation buffer [150 mM NaCl, 100 mM Tris-HCl (pH 8), 1% NP-40, protease and phosphatase inhibitors] by gently pipetting. Protein A/G-magnetic beads (Thermo Fisher Scientific, Waltham, MA) were incubated with 2 μg of mouse anti-KPC1 IgG for 2 hours at 4°C in a rotator. In all the conditions, the beads were washed three times with the washing buffer [150mM NaCl, 100 mM Tris-HCl (pH 8)] on a magnetic rack, and then incubated overnight with 800 μg of whole cell lysate at 4°C with a rotator. The beads were washed 3X times with the immunoprecipitation buffer and then boiled in the protein loading buffer for 5 min at 95°C in a dry bath. For ubiquitin-based IP, MM cells were washed twice with ice-cold PBS and lysed on ice for 30 min in Lysis buffer [10 mM Tris-Cl pH 7.5, 150 mM NaCl, 0.5 mM EDTA, 0.5 % NP-40]. Lysates were clarified by centrifugation at 17,000 × g for 10 min at 4 °C and then diluted with fresh lysis buffer lacking detergent. ChromoTek Ubiquitin-Trap Magnetic Agarose beads (Proteintech, Chicago, IL) (25 µL slurry per sample) were equilibrated by three washes in the same buffer, then incubated with 500 µL of the diluted lysate for 1 hour at 4 °C with end-over-end mixing. Beads were washed three times in wash buffer [10 mM Tris-Cl pH 7.5, 150 mM NaCl, 0.05 % NP-40, 0.5 mM EDTA] to remove non-specific binders. Bound proteins were eluted in sample buffer by heating at 95 °C for 5 min. The results are representative of three independent experiments (n = 3).

**Multiplex immunofluorescence**

Paraffin-embedded tissue microarrays (TMA) for AJCC stage III and IV melanoma established at Saint John’s health center included MM cases of AJCC stage III or IV, consisting of 41 cases of stage IV metastatic and 69 cases of stage III metastatic tumor tissues. TMA were clinically well-annotated and patients have greater than 5-years follow-up. All mIF were performed as previously described ^4^ using Opal 7-Color Manual IHC Kit (NEL 811001KT, Akoya Biosciences, Marlborough, MA). Detailed information about primary antibodies, the panels, and the corresponding fluorophores are indicated in Table S1. The images of multiplex-stained slides were acquired by the Mantra Multispectral Imaging System (v1.0, Akoya Biosciences, Marlborough, MA). Filter cubes used for multispectral imaging were DAPI (440–680 nm), fluorescein isothiocyanate (FITC, 520–680 nm), and Cy3 (570–690 nm), Texas Red (580–700 nm), and Cy5 (670–720 nm). The normalization of the signal intensities was performed for each marker and spectral unmixing was conducted by InForm Analysis software (v.2.6.0, Akoya Biosciences, Marlborough, MA). For the quantification of the targeted protein levels, positive cells were counted as previously described ^5^. The cells co-expression MART1 were selected for analysis. Two to three images for each core in the TMA slide were captured at 40X magnification and the average values were defined as the expression for patients. The median expression of the specified marker in each sample were compared for statistical significance.

**Wound healing assay**

For siRNA-mediated *KPC1*-knockdown experiments, cells were plated on 6-well plates at a density of 3 × 10^5^ cells/well. After cells grew to 80%-90% confluency, vertical scratches were generated in the cell layer using 1000 μL pipette tips. Cells were then transfected with siKPC1 or negative-control siRNA in triplicate. Photographs of the wound area were taken at 0, 24, 48 and 72 hours after scratching to calculate the cell migration rate.

**Analysis of scRNA-seq dataset**

The R package Seurat version 4.3 ^6^ was used to re-analyze GSE115978 ^7^. The cited gene expression profiles were converted to Seurat objects by R version 4.4.2. The scRNA-seq data was first log-normalized. Then, the data was further scaled using the “ScaleData” function and highly variable genes were extracted by performing the “FindVariableFeatures” function. Principal component analysis (PCA) was performed to decrease dimensionality and “FindClusters” functions and “FindNeighbors” were conducted to re-cluster cells into subgroups using parameters of dim = 10 and resolution = 0.5. The subgroups of cells were visualized by t-SNE plot using the “RunTSNE” function.

**Cycloheximide chasing assay**

HM-0525 or DP-0574 cells (2.5 × 10^5^ cell/well) were transiently transfected with 25 nM siRNA (Dharmacon, Lafayette, CO) using jet PRIME (VWR International). Cycloheximide (50 μg/ml, Sigma-Aldrich, St. Louis, MO) was added 6, 4, and 2 hours before the 72-hour time point, and all samples were collected at 72 hours after transfection for protein extraction and Western blot analysis. CHX chase experiments were performed in three independent biological experiments (n = 3).

**MG132 treatment**

MM cells were seeded, and MG132 (10 μM, Selleck Chemicals LLC, Houston, TX) was added for 1, 2, and 4 hours before sample collection. All samples were collected for protein extraction and Western blot analysis. MG132 treatments were performed in three independent biological experiments (n = 3).

**In vitro TGF-β treatment**

MM cells (HM-0525 and DP-0574) were seeded (5.0 × 10^5^ cell/well) in a tissue culture treated 60 mm dish in complete culture medium. After 24 hours, complete culture medium containing TGF-β1 (10 ng/mL, 7754-BH-005, R&D Systems, Minneapolis, MN) was replaced and incubated for 48 hours before sample collection. All samples were collected for protein extraction and Western blot analysis. The experiments were performed in three independent biological experiments (n = 3).

**Small interference RNA**

Briefly, HM-0525 and DP-0574 cells (2.5 × 10^5^ cell/well) were transfected with 25 nM ON-TARGET plus SMART pool siRNA to downregulate human *KPC1* or non-targeting pool siRNA as a control (Dharmacon, Lafayette, CO) using jet PRIME (VWR International). The mRNA and protein levels were analyzed 72 hours after transfection by RT-qPCR and Western blot to confirm KPC1 knockdown. Other siRNA reagents used to target different molecules throughout the manuscript are listed in Table S1.

**Pathway analysis**

Single-sample gene set enrichment analysis (ssGSEA) ^8^ were performed to calculate the pathway enrichment score. The melanoma specific mRNA expression profiles obtained from deconvolution analysis were used for calculating the enrichment scores for all pathways included in the “h.all.v2023.2.Hs.symbols.gmt” file.

**References**

1. Kobayashi Y, Bustos MA, Hayashi Y, Yu Q, Hoon D. Interferon-induced factor 16 is essential in metastatic melanoma to maintain STING levels and the immune responses upon IFN-γ response pathway activation. *J Immunother Cancer* 2024, **12**(10).

2. Zhang X, Bustos MA, Gross R, Ramos RI, Takeshima TL, Mills GB*, et al.* Interleukin enhancer-binding factor 2 promotes cell proliferation and DNA damage response in metastatic melanoma. *Clin Transl Med* 2021, **11**(10)**:** e608.

3. Schneider CA, Rasband WS, Eliceiri KW. NIH Image to ImageJ: 25 years of image analysis. *Nature Methods* 2012, **9**(7)**:** 671-675.

4. Gopi LK, Kidder BL. Integrative pan cancer analysis reveals epigenomic variation in cancer type and cell specific chromatin domains. *Nat Commun* 2021, **12**(1)**:** 1419.

5. Furuhashi S, Bustos MA, Mizuno S, Ryu S, Naeini Y, Bilchik AJ*, et al.* Spatial profiling of cancer-associated fibroblasts of sporadic early onset colon cancer microenvironment. *NPJ Precis Oncol* 2023, **7**(1)**:** 118.

6. Hao Y, Hao S, Andersen-Nissen E, Mauck WM, 3rd, Zheng S, Butler A*, et al.* Integrated analysis of multimodal single-cell data. *Cell* 2021, **184**(13)**:** 3573-3587.e3529.

7. Jerby-Arnon L, Shah P, Cuoco MS, Rodman C, Su MJ, Melms JC*, et al.* A Cancer Cell Program Promotes T Cell Exclusion and Resistance to Checkpoint Blockade. *Cell* 2018, **175**(4)**:** 984-997.e924.

8. Barbie DA, Tamayo P, Boehm JS, Kim SY, Moody SE, Dunn IF*, et al.* Systematic RNA interference reveals that oncogenic KRAS-driven cancers require TBK1. *Nature* 2009, **462**(7269)**:** 108-112.

**Supplementary Figures**


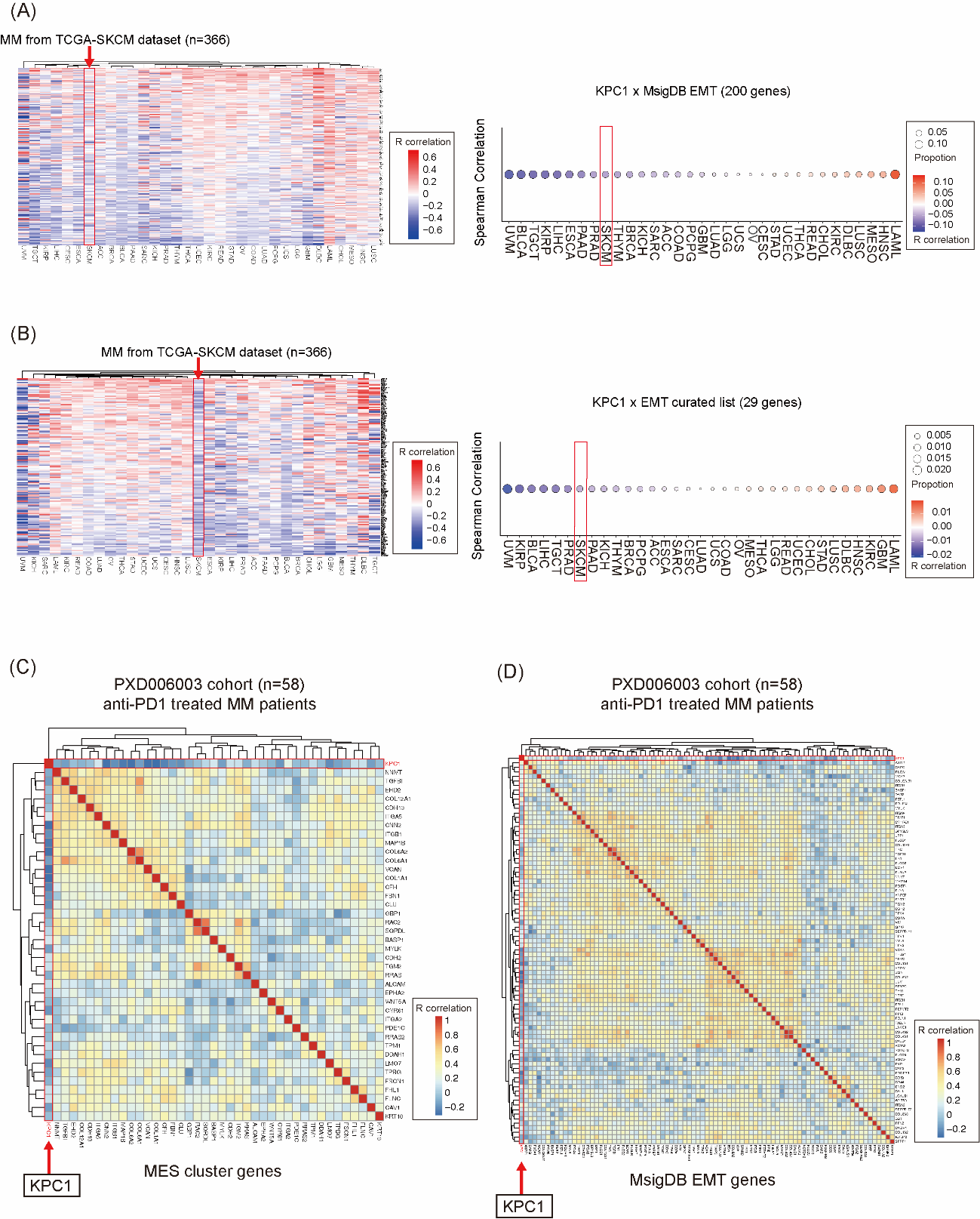


**Supplementary Figure 1. Correlation between KPC1 expression and MES markers.** (A-B) Left: Heatmap of Spearman correlation between *KPC1* and 200 MsigDB EMT (A) and 29 EMT curated list (B) across 33 TCGA tumor types. The x-axis represents the cancer cohorts, and the y-axis represents the genes within the EMT-related gene sets; a red arrow highlights SKCM. Right: Dot plot of Spearman R values for KPC1 versus these gene sets in each tumor type; circle size indicates the fraction of significant genes. (C-D) Heatmap depicting the correlation between KPC1 expression and EMT-related genes ((C) MES cluster gene set, (D) MSigDB EMT gene set) in the PXD06003 cohort. Cohort including patients treated with anti-PD-1. Both x-axis and y-axis represent gene names, with the heatmap displaying co-expression R values. Red indicates positive correlation, and blue indicates negative correlation. Correlation was calculated using Spearman’s rank correlation coefficient.


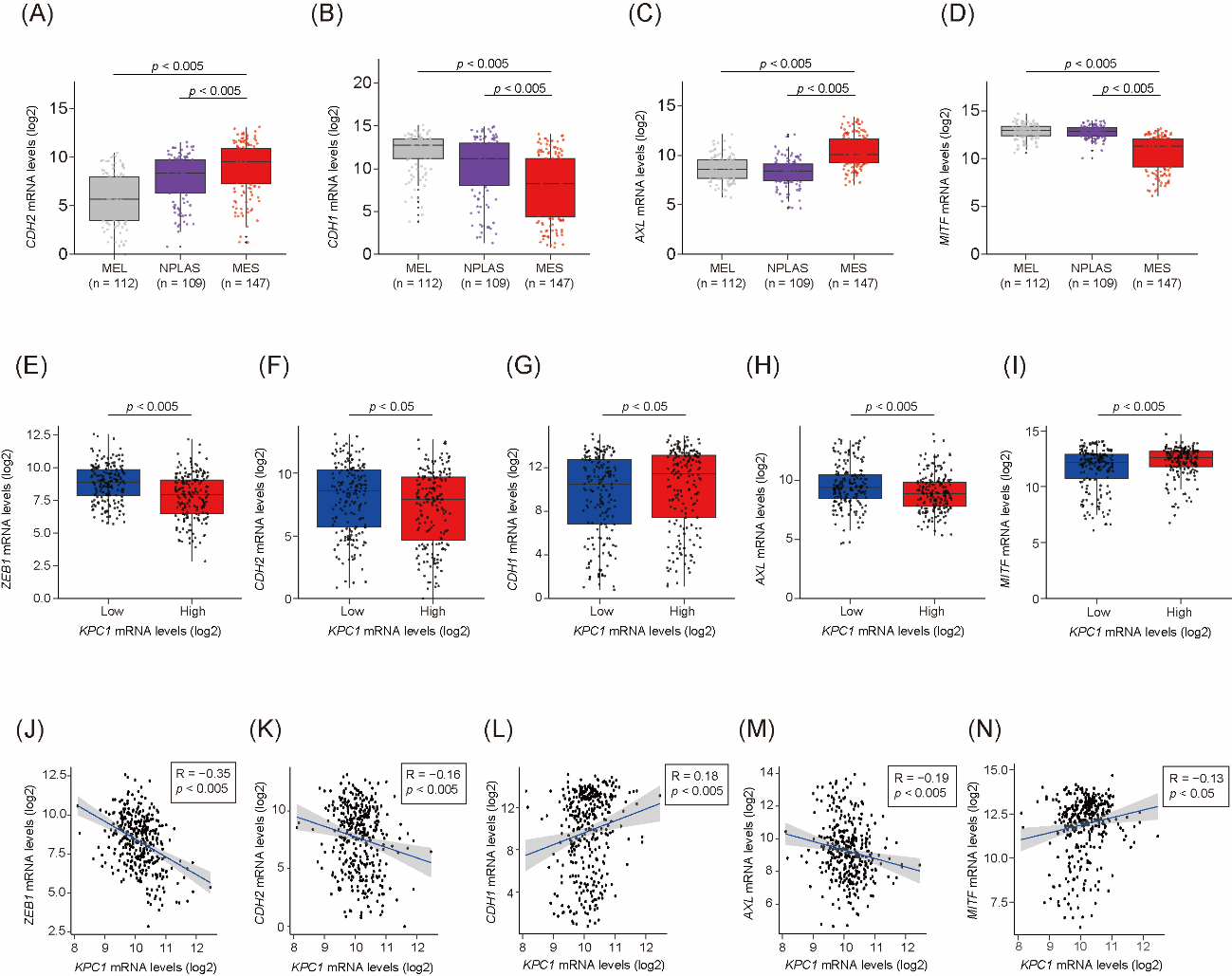


**Supplementary Figure 2.** ***KPC1* expression levels are associated with MES gene signatures in MM of the TCGA-SKCM dataset.** (A-D) Box plot comparing *CDH2, CDH1, AXL, and MITF* expression among the MEL, NPLAS, and MES clusters. (E-I) Box plot comparing the expression levels of *ZEB1, CDH2*, *CDH1, AXL, and MITF* between high- and low-*KPC1* expression groups in metastatic melanoma (MM) from the TCGA-SKCM cohort. (J-N) Dot plot showing the correlation between *KPC1* expression and *ZEB1, CDH2, CDH1, AXL*, and *MITF* expression. Correlation was assessed using Spearman’s rank correlation coefficient. Statistical significance for three-group comparisons was determined using one-way ANOVA followed by Tukey’s post hoc test, while two-group comparisons were assessed using the Mann-Whitney U test.


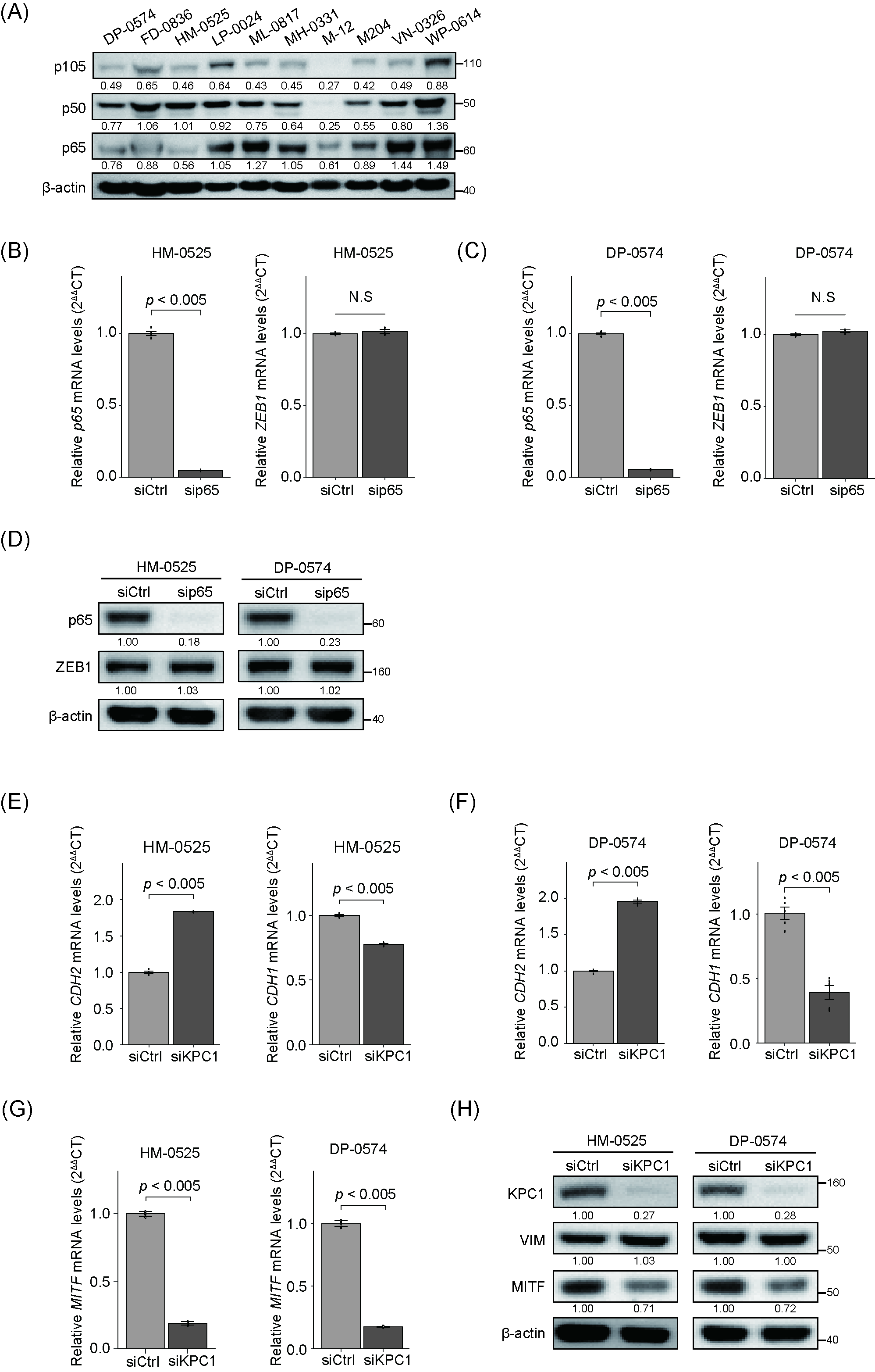


**Supplementary Figure 3. KPC1 silencing induces a cadherin switch and MITF reduction, while p65 knockdown does not affect ZEB1 expression.** (A) Baseline expression of NF-κB1 subunits across ten metastatic melanoma cell lines. Whole-cell lysates were analyzed by Western blot for p105, p50, and p65, with β-actin as loading control. (B-C) qRT-PCR analysis of *ZEB1* and *p65* mRNA in HM-0525 (B) and DP-0574 (C) cells transfected with sip65. (D) Western blot analysis of p65 and ZEB1 following siRNA-mediated silencing of sip65 in HM-0525 and DP-0574 cell lines. (E-F) qRT-PCR of *CDH1* (E) and *CDH2* (F) mRNA in HM-0525 and DP-0574 cell lines transfected with siKPC1. (G) qRT-PCR analysis of *MITF* mRNA in HM-0525 and DP-0574 cell lines transfected with siKPC1. (H) Western blot analysis of KPC1, VIM and MITF following siRNA-mediated silencing of KPC1 in HM-0525 and DP-0574 cell lines. Data are presented as the mean ± standard deviation. Statistical significance between groups was assessed using a two-tailed unpaired Student's t-test. These data represent three independent experiments, each carried out in triplicate.


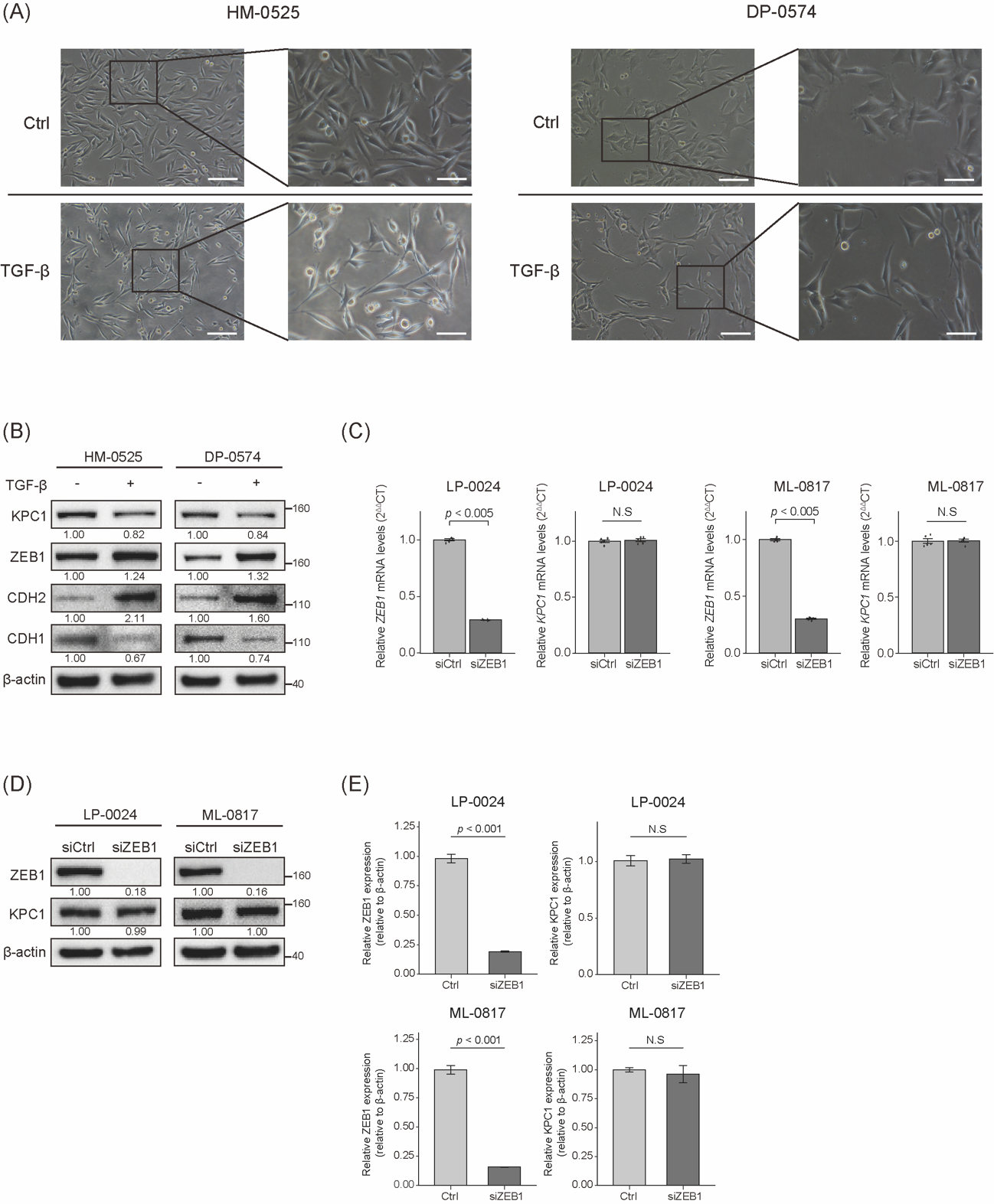


**Supplementary Figure 4. Exogenous induction of a MES-like program in melanoma cells and its impact on KPC1 and MES markers.**

**(**A) Phase-contrast images of HM-0525 and DP-0574 cells under control (Ctrl) or TGF-β exposure (Scale bars; 100 µm (left), 50 µm (right)). (B) Western blot analysis of KPC1, ZEB1, CDH2, and CDH1 following TGF-β in HM-0525 and DP-0574 cell lines. (C) qRT-PCR of *ZEB1* and *KPC1* mRNA in LP-0024 and ML-0817 cell lines transfected with siZEB1. (D) Western blot analysis of ZEB1 and KPC1 following siRNA-mediated silencing of siZEB1 in LP-0024 and ML-0817 cell lines. (E) Quantification of Western blot images corresponding to Figure 4D (LP-0024 and ML-0817). Band intensities were normalized to β-actin and expressed relative to the matched control within each cell line. Data are presented as the mean ± standard deviation. Statistical significance between groups was assessed using a two-tailed unpaired Student's t-test. These data represent three independent experiments, each carried out in triplicate.


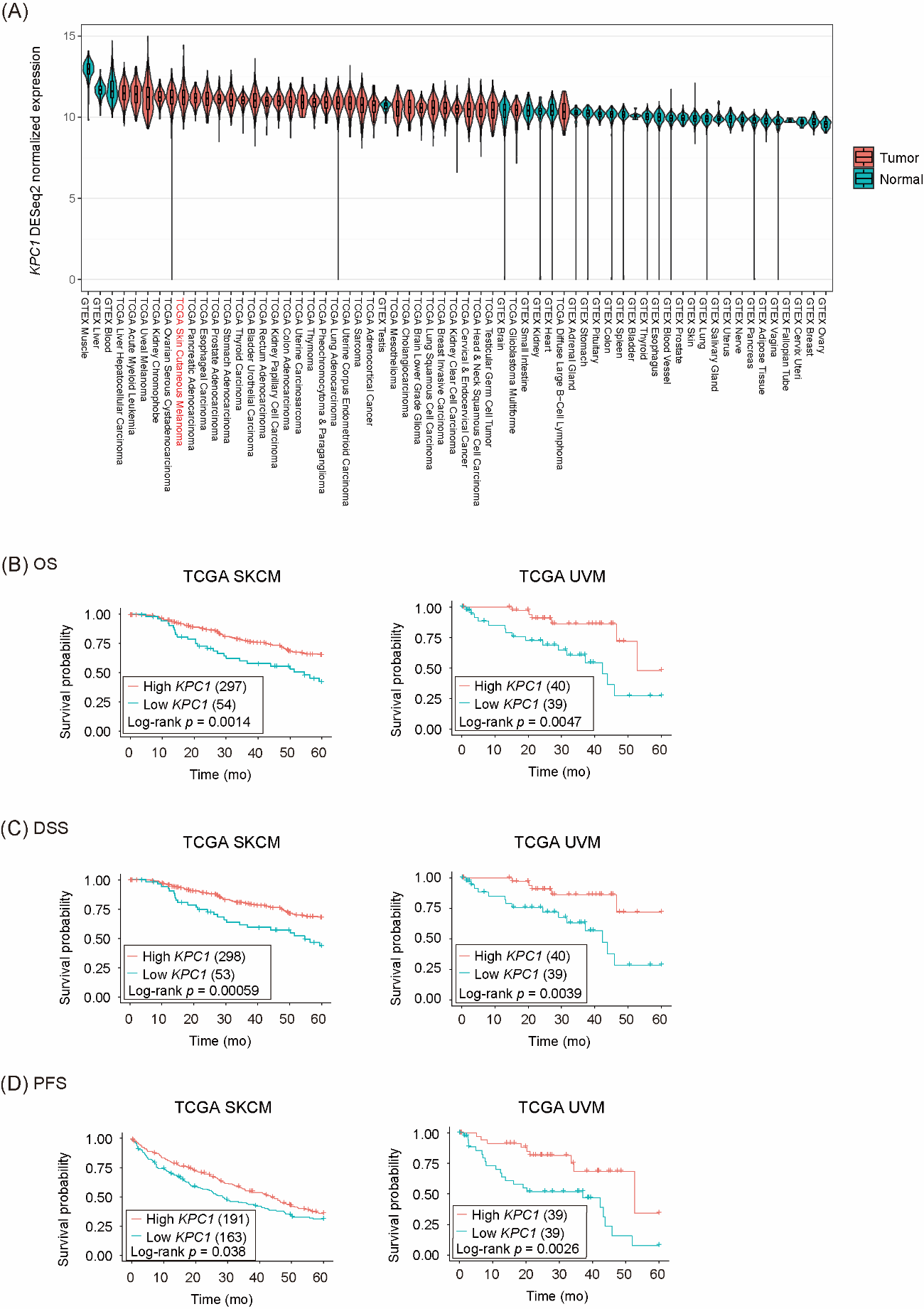


**Supplementary Figure 5. *KPC1* mRNA levels have a prognostic significance across multiple cancer types. (**A) *KPC1* mRNA levels in tumor tissues (TCGA dataset) and normal tissues (GTEx dataset). Statistical differences between normal and tumor tissues were evaluated using the Mann-Whitney U test. (B-D) Patients were stratified into high- and low-*KPC1* expression level groups to evaluate the prognostic impact of *KPC1* expression levels in overall survival (OS, B), disease-specific survival (DSS, C), and progression-free survival (PFS, D) across cancer types.


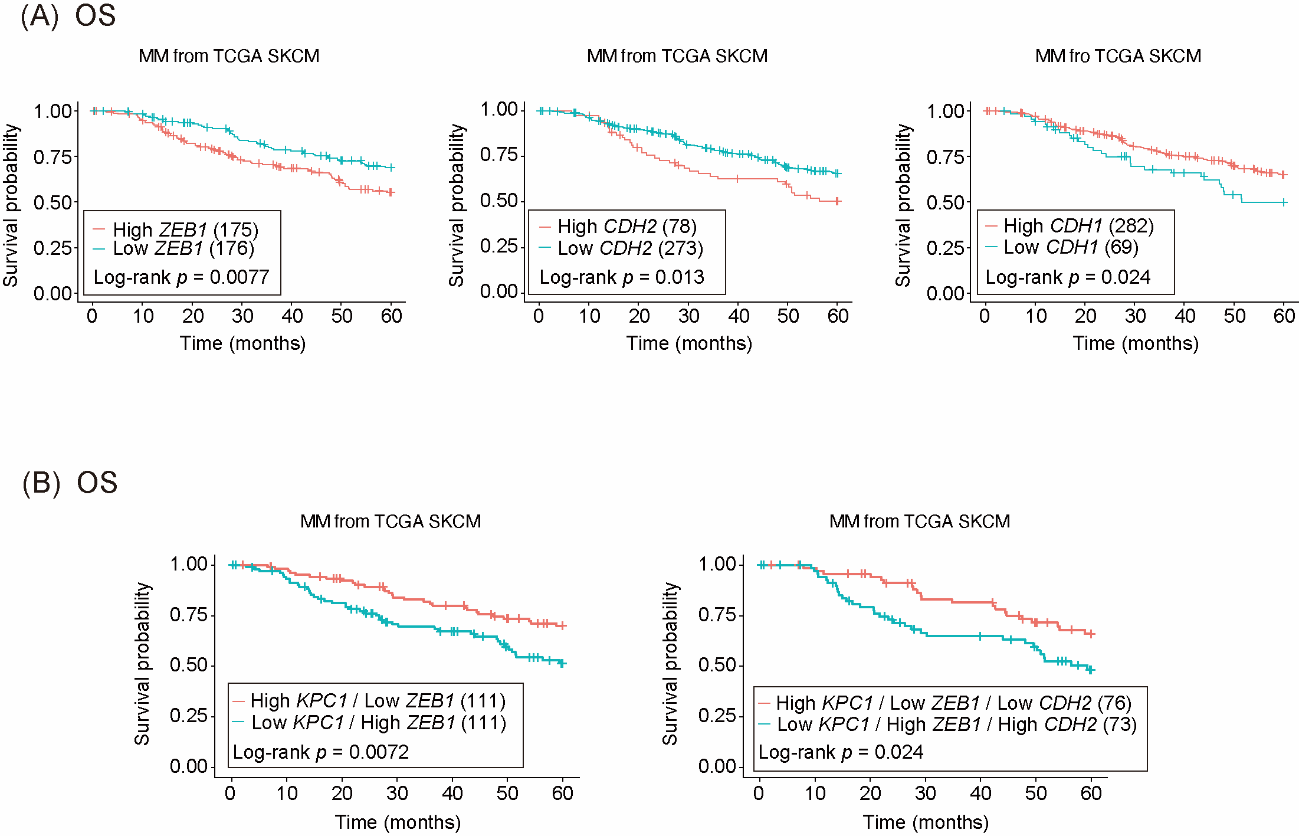


**Supplementary Figure 6. Kaplan-Meier survival analysis of overall survival (OS) in MM patients based on *ZEB1*, *CDH1*, *CDH2*, and *KPC1* expression in the TCGA-SKCM dataset.** (A) OS analysis based on individual gene expression levels. (left) high and low *ZEB1* expression, (middle) high and low *CDH2* expression, and (right) high and low *CDH1* expression. (B) OS analysis integrating *KPC1*, *ZEB1*, and *CDH2* expression levels. (left) Kaplan-Meier curves comparing OS between *KPC1* high / *ZEB1* low and *KPC1* low / *ZEB1* high. (right) OS analysis based on combined expression of *KPC1*, *ZEB1*, and *CDH2* (*KPC1* high / *ZEB1* low / *CDH2* low vs. *KPC1* low / *ZEB1* high / *CDH2* low.


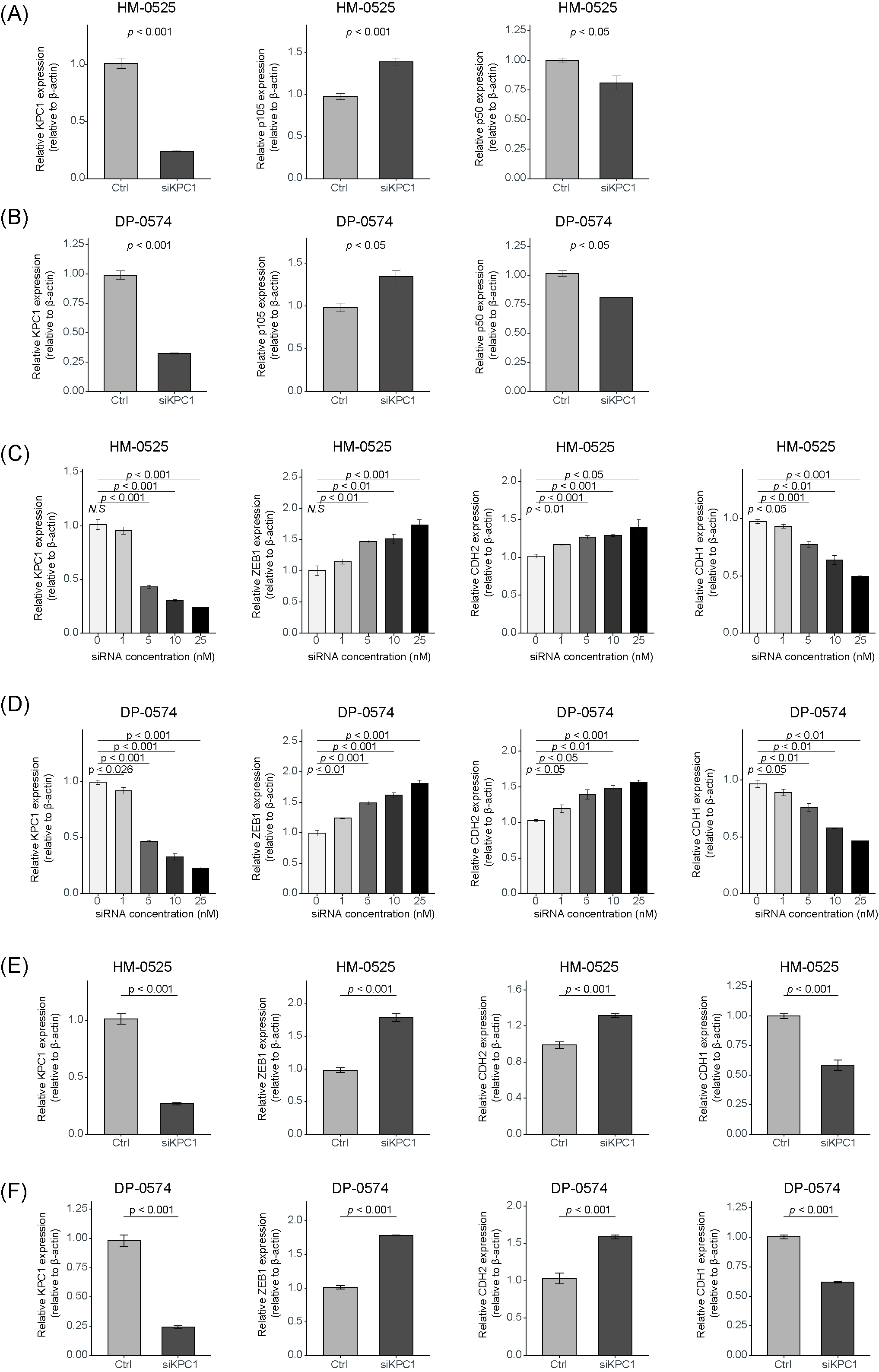


**Supplementary Figure 7. Quantitative immunoblot analyses and statistics for Figure 4. (**A-F) Quantification of Western blot images corresponding to Figure 4A (HM-0525, A), corresponding to Figure 4A (DP-0574, B), corresponding to Figure 4G (HM-0525, C), corresponding to Figure 4H (DP-0574, D), corresponding to Figure 4I (HM-0525, E), corresponding to Figure 4I (DP-0574, F). Band intensities were normalized to β-actin and expressed relative to the matched control within each cell line. Bars show mean ± SD from n = 3 independent biological experiments. Statistics: two-sided Welch’s t-tests were used for pairwise comparisons in panels A, B, E, and F. For titrations in panels C and D, each dose was compared with 0 using two-sided Welch’s t-tests with Benjamini–Hochberg adjustment for multiple comparisons. Exact p values are annotated on the plots.


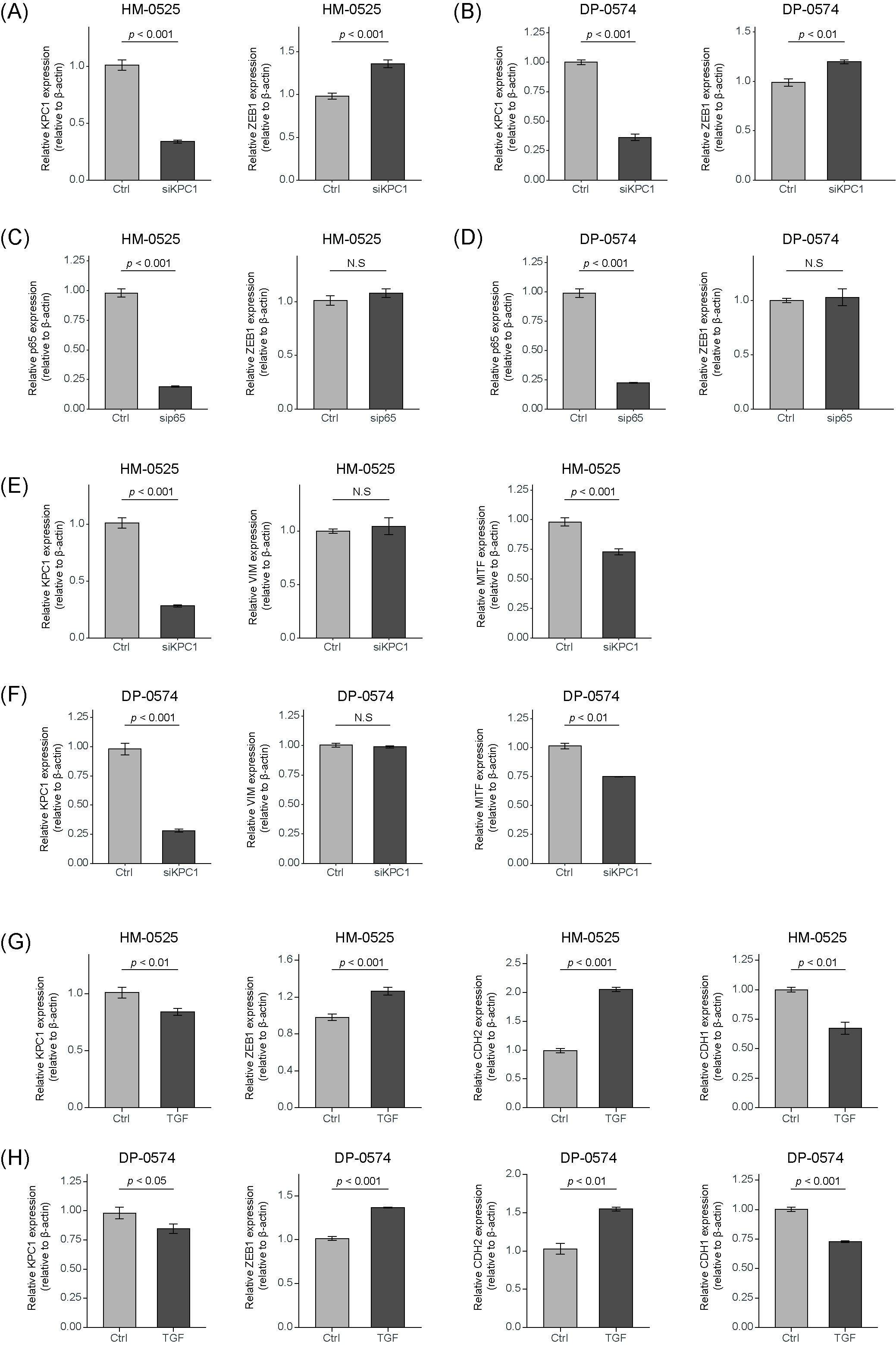


**Supplementary Figure 8. Quantitative immunoblot analyses and statistics for Figure 5F, Supplementary Figure 3D, 3H, and 4B. (**A-H) Quantification of Western blot images corresponding to Figure 5F (HM-0525, A), corresponding to Figure 5F (DP-0574, B), corresponding to Supplementary Figure 3D (HM-0525, C), corresponding to Supplementary Figure 3D (DP-0574, D), corresponding to Supplementary Figure 3H (HM-0525, E), corresponding to Supplementary Figure 3H (DP-0574, F), corresponding to Supplementary Figure 4B (HM-0525, G), corresponding to Supplementary Figure 4B (DP-0574, H). Band intensities were normalized to β-actin and expressed relative to the matched control within each cell line. Bars show mean ± SD from n = 3 independent biological experiments. Statistics: two-sided Welch’s t-tests were used for pairwise comparisons. Exact p values are annotated on the plots.
