# Supplementary material for: Ubiquitin E3 ligase KPC1 governs mesenchymal metastatic melanoma reprogramming via proteasomal degradation of ZEB1
Source: Cell Death Dis. 2025 Dec 22;16(1):897. doi: 10.1038/s41419-025-08262-z (PMC12722757; doi:10.1038/s41419-025-08262-z)
Supplement: Supplementary file 4 — Supplementary Materials WB uncropped [file 41419_2025_8262_MOESM4_ESM.docx]

**Supplementary Materials** for

**Ubiquitin ligase KPC1 governs mesenchymal reprogramming via proteasomal degradation of ZEB1**

Yusuke Nakano *et al*.

*Corresponding author. E-mail: [dave.hoon@providence.org](http://dave.hoon@providence.org)


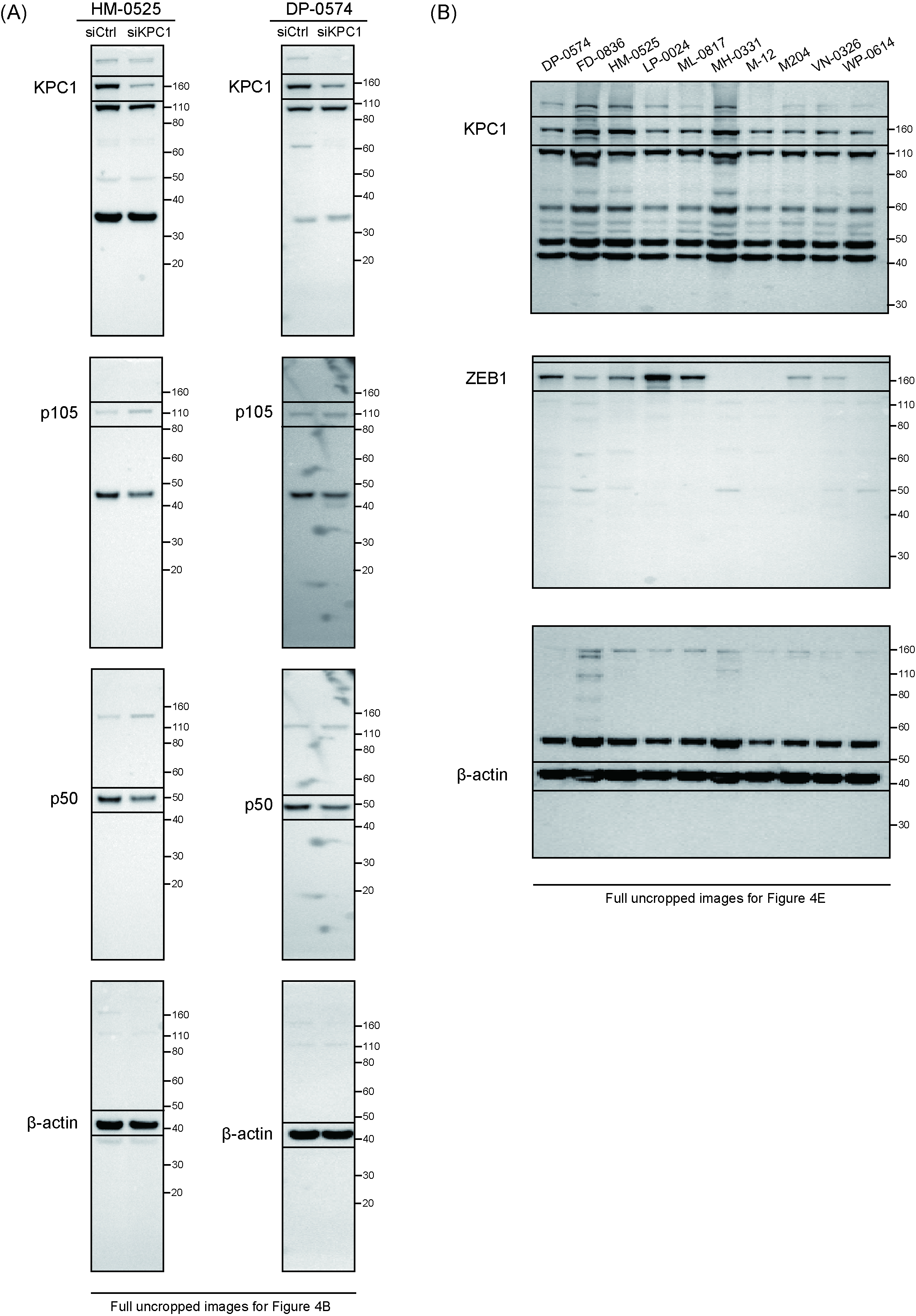


Uncropped western blot images for figure 4B and 4E.


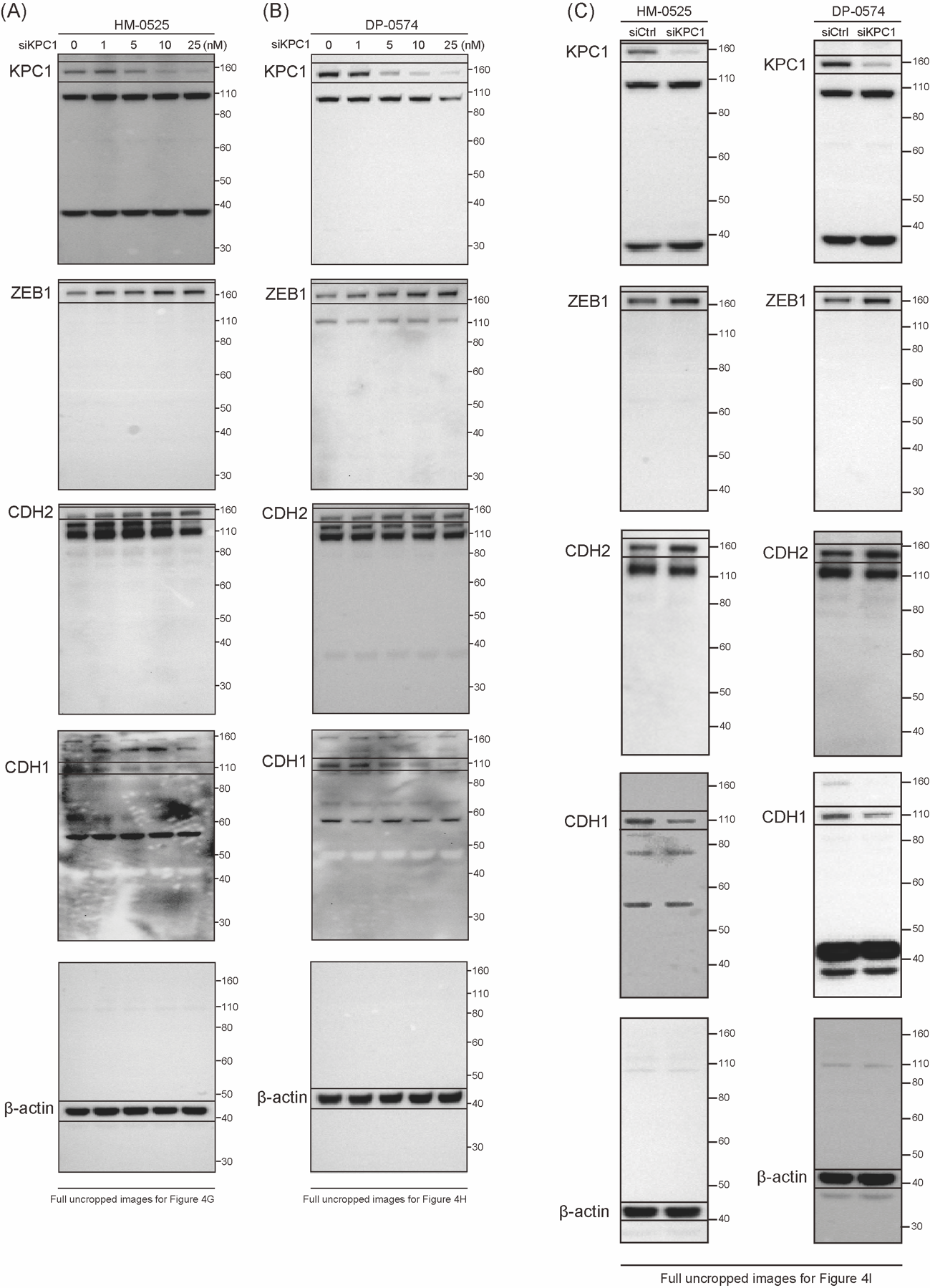


Uncropped western blot images for figure 4G, 4H, and 4I.


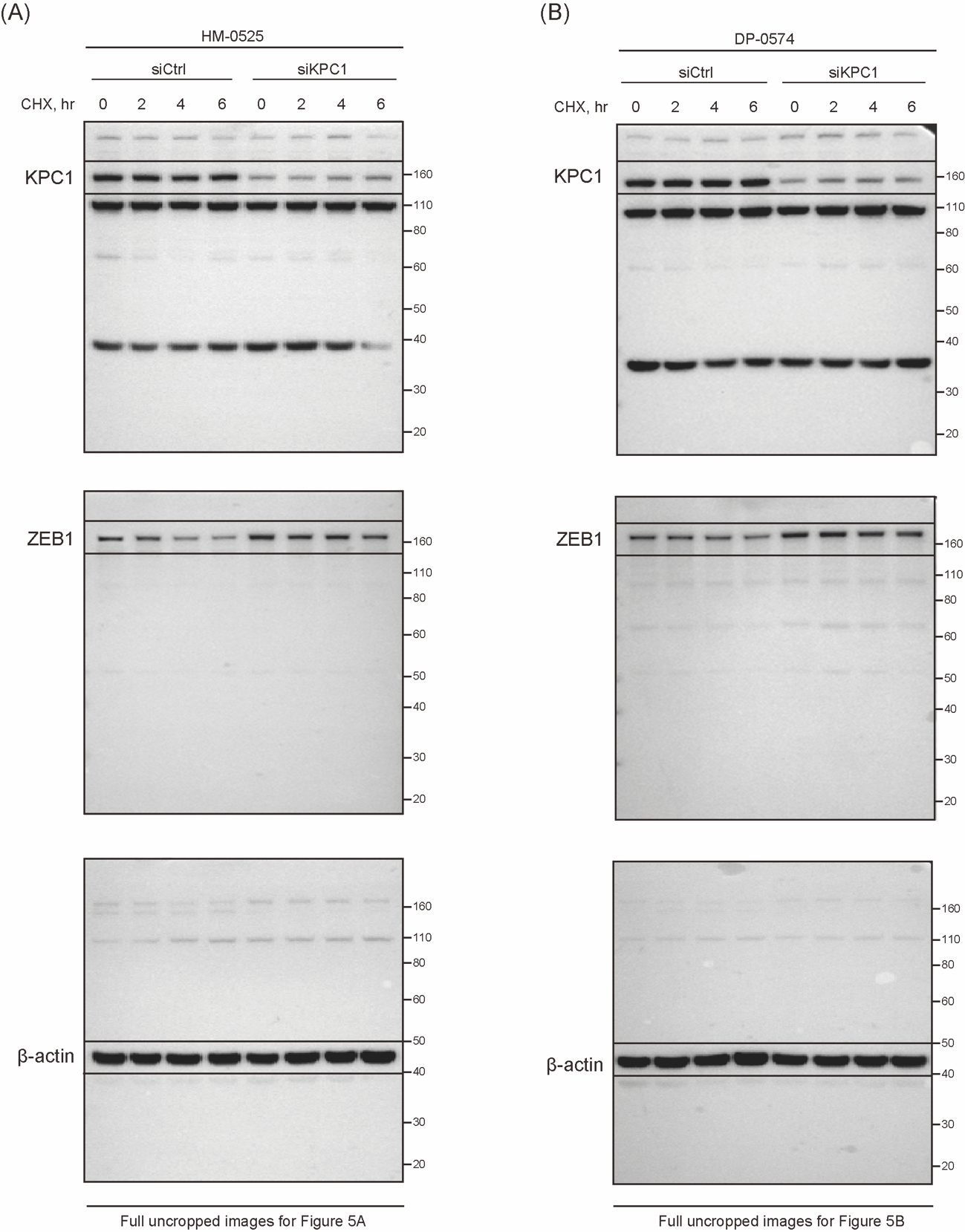


Uncropped western blot images for figure 5A and 5B.


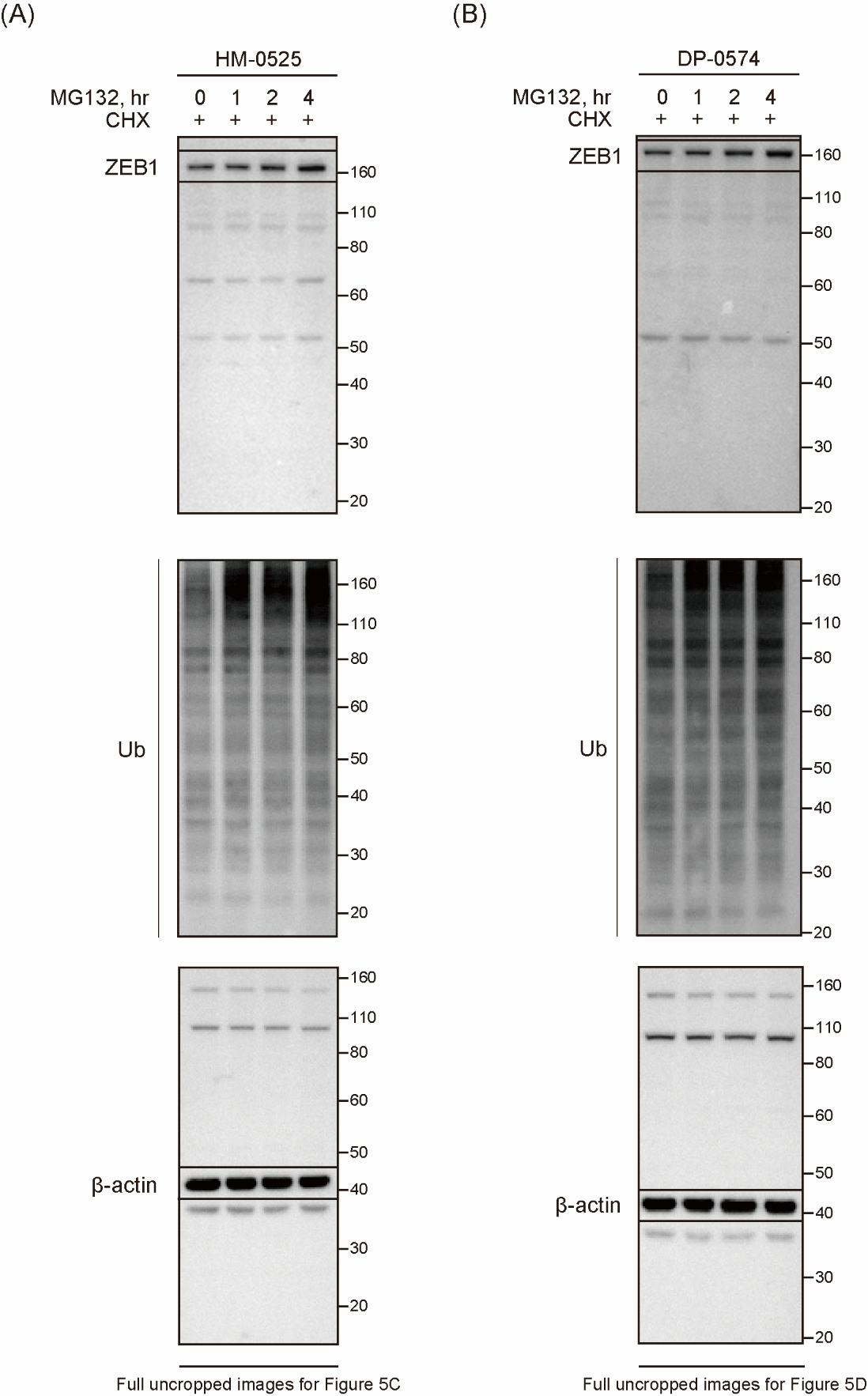


Uncropped western blot images for figure 5C and 5D.


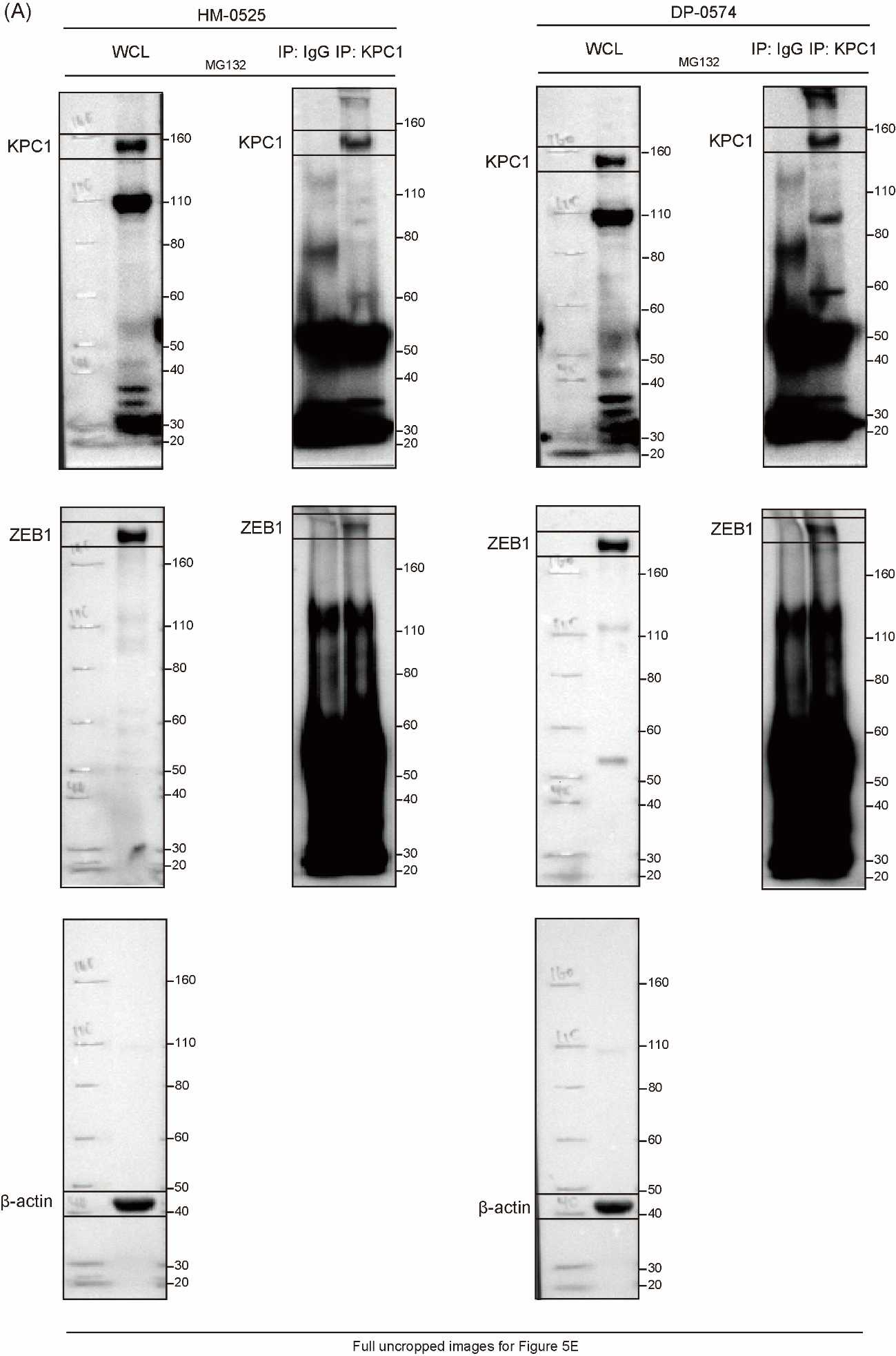
 Uncropped western blot images for figure 5E.


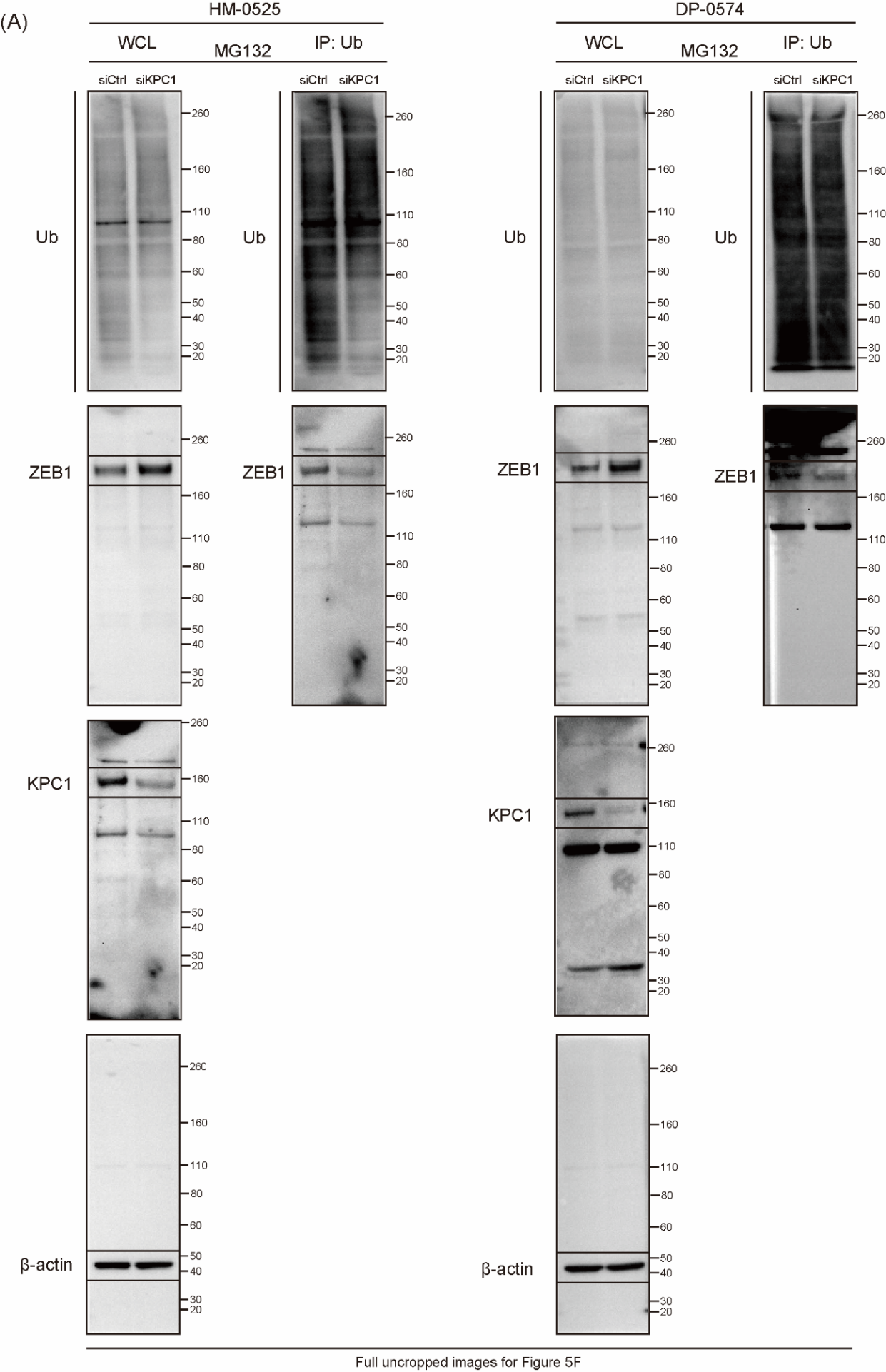
 Uncropped western blot images for figure 5F.


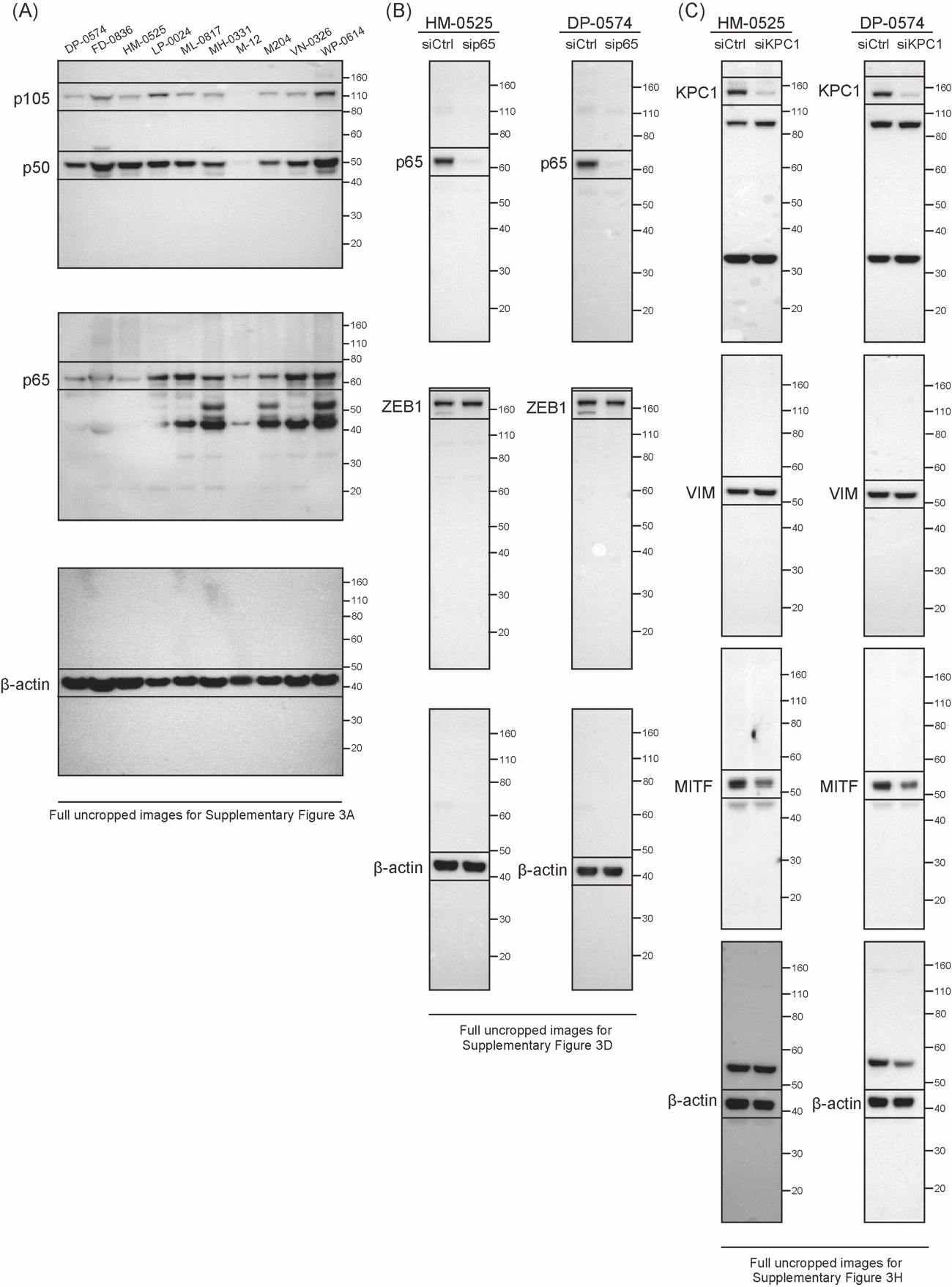


Uncropped western blot images for supplementary figure 3A ,3D, and 3H.


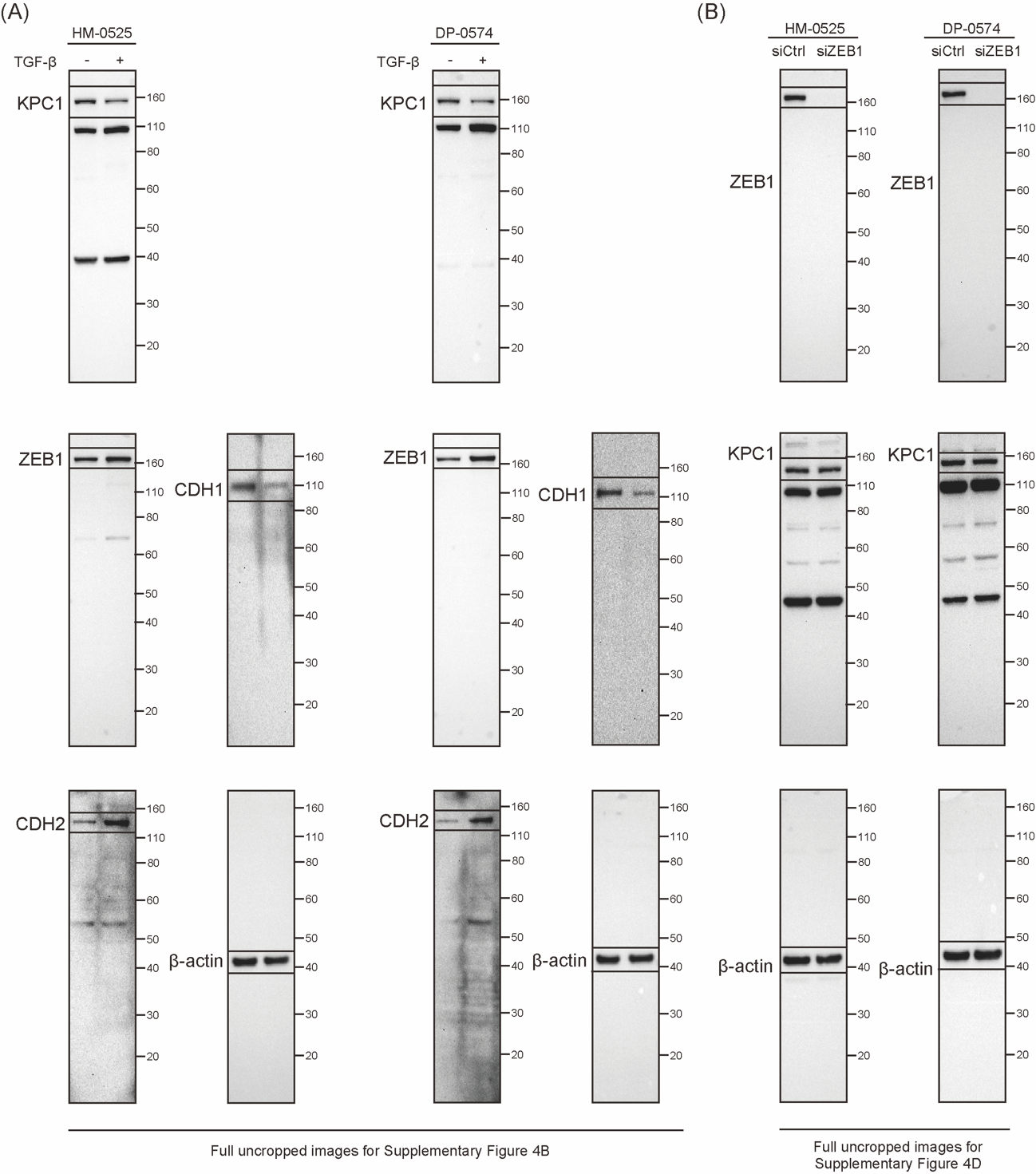


Uncropped western blot images for supplementary figure 4B and 4D.
